# Morusin Alleviates Aortic Valve Calcification by Inhibiting Valve Interstitial Cell Senescence Through Ccnd1/Trim25/Nrf2 Axis

**DOI:** 10.1002/advs.202307319

**Published:** 2024-03-19

**Authors:** Zongtao Liu, Kan Wang, Chen Jiang, Yuqi Chen, Fayuan Liu, Minghui Xie, Wai Yen Yim, Dingyi Yao, Xingyu Qian, Shiqi Chen, Jiawei Shi, Kang Xu, Yixuan Wang, Nianguo Dong

**Affiliations:** ^1^ Department of Cardiovascular Surgery Union Hospital Tongji Medical College Huazhong University of Science and Technology Wuhan 430022 China; ^2^ Hubei Provincial Engineering Technology Research Center for Chinese Medicine Processing School of Pharmacy Hubei University of Chinese Medicine Wuhan 430065 China; ^3^ Hubei Shizhen Laboratory Wuhan 430065 China; ^4^ Key Laboratory of Organ Transplantation Ministry of Education NHC Key Laboratory of Organ Transplantation Key Laboratory of Organ Transplantation Chinese Academy of Medical Sciences Wuhan 430022 China

**Keywords:** antiaging, calcific aortic valve disease, molecular target, natural product

## Abstract

The senescence of aortic valve interstitial cells (VICs) plays a critical role in the progression of calcific aortic valve disease (CAVD). However, the precise mechanisms underlying the senescence of VICs remain unclear, demanding the identification of a novel target to mitigate this process. Previous studies have highlighted the anti‐aging potential of morusin. Thus, this study aimed to explore the therapeutic potential of morusin in CAVD. Cellular experiments reveal that morusin effectively suppresses cellular senescence and cause a shift toward osteogenic differentiation of VICs in vitro. Mechanistically, morusin activate the Nrf2‐mediated antiaging signaling pathway by downregulating CCND1 expression and aiding Keap1 degradation through Trim 25. This activation lead to the upregulated expression of antioxidant genes, thus reducing reactive oxygen species production and thereby preventing VIC osteogenic differentiation. In vivo experiments in ApoE^−/−^ mice on a high‐fat Western diet demonstrate the positive effect of morusin in mitigating aortic valve calcification. These findings emphasize the antiaging properties of morusin and its potential as a therapeutic agent for CAVD.

## Graphical abstract

scRNA‐seq reveals that senescence of aortic valve interstitial cells (VICs) plays a critical role in the progression of calcific aortic valve disease (CAVD). Morusin activates antiaging signaling in VICs, reducing oxidative stress and preventing calcification. This suggests morusin may be a potential therapeutic agent for calcific aortic valve disease.

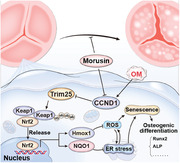

## 1 Introduction

Calcific aortic valve disease (CAVD) is increasingly prevalent among the elderly, with its incidence increasing in tandem with the growing number of older individuals.^[^ ^1^ ^]^ Currently, none of the currently available medical interventions effectively halt CAVD progression, leaving surgical or transcatheter aortic valve replacement as the only option for patients experiencing severe symptomatic CAVD.^[^ ^2^ ^]^ A correlation exists between valvular calcification and chronic inflammation as well as osteogenic differentiation.^[^ ^3^, ^4^ ^]^ Aortic valve interstitial cells (VICs), which are the primary cellular components in aortic valves,^[^ ^5^ ^]^ are believed to play a crucial role in CAVD development.^[^ ^6^, ^7^ ^]^ However, the detailed molecular pathways and osteogenic mechanisms of CAVD are not fully understood. Therefore, a comprehensive investigation into the pathogenesis of CAVD and the identification of potential therapeutic targets is essential to slow its progression.

CAVD is a chronic condition characterized by inflammatory infiltration, aortic valve thickening, and fibrosis matrix formation.^[^ ^8^ ^]^ The initial phase of CAVD begins with endothelial damage leading to the infiltration of inflammatory cells into the valvular matrix. This creates a pro‐inflammatory and pro‐osteogenic environment.^[^ ^9^, ^10^ ^]^ VICs, in the presence of inflammatory molecules, gradually adopt osteoblast‐like properties, contributing to leaflet remodeling and valve mineralization.^[^ ^11^ ^]^ Genetic studies have uncovered the upregulated expression of bone morphogenetic protein 2 (BMP2), alkaline phosphatase (ALP), and runt‐related transcription factor 2 (RUNX2) in osteogenic VICs, along with the involvement of multiple intracellular pathways such as the NOTCH, WNT‐β‐catenin, and NF‐κB pathways.^[^ ^8^, ^12^ ^]^ Given the mechanisms of CAVD, identifying actionable targets is crucial to effectively prevent the progression of valvular calcification.

Cellular senescence may have a significant effect on the advancement of cardiac pathologies.^[^ ^13^ ^]^ Cellular senescence refers to a permanent state of cell cycle arrest triggered by diverse stressors, such as DNA damage, reactive oxygen species (ROS), aging, or mitochondrial dysfunction.^[^ ^14^, ^15^ ^]^ Senescent cells influence the cellular microenvironment by secreting biologically active substances such as β‐galactosidase, matrix metalloproteinases (MMPs), and inflammatory cytokines. Proteins related to cell cycle arrest, particularly p16 and p53, have shown atypical expression in senescent cells, potentially serving as molecular indicators of cellular senescence.^[^ ^13^ ^]^ Senescence of VICs likely plays a crucial role in CAVD's pathophysiology. Thus, targeting senescent VICs could be an effective strategy for addressing age‐related CAVD.

Numerous phytochemicals and natural products demonstrate various pharmacological properties,^[^ ^16^, ^17^, ^18^, ^19^, ^20^ ^]^ including antiaging,^[^ ^21^ ^]^ antioxidant,^[^ ^22^ ^]^ antitumor,^[^ ^23^ ^]^ and anti‐inflammatory activities.^[^ ^24^ ^]^ Morusin, a prenylated flavone, can extend lifespan in *Caenorhabditis elegans* worms^[^ ^25^ ^]^ and displays anti‐inflammatory, antiaging, antitumor, and antioxidative effects, suggesting promising therapeutic potential for a wide range of chronic diseases.^[^ ^26^, ^27^ ^]^ It exhibits antitumor effects in breast cancer, melanoma, gastric, renal cell carcinoma, glioblastoma, and pancreatic cancer.^[^ ^28^, ^29^, ^30^, ^31^ ^]^ Additionally, morusin exerts therapeutic efficacy in inflammatory diseases including chondrocyte inflammation and osteoarthritis.^[^ ^32^ ^]^ Morusin modulates intracellular pathways such as Wnt/β‐catenin, NF‐κB, and MAPK signaling.^[^ ^29^ ^]^ Mulberry leaf extracts have therapeutic potential for cardiovascular disease and diabetes,^[^ ^33^, ^34^ ^]^ yet clinical trials for morusin are lacking. Based on these findings, this study hypothesized that morusin might impede VIC senescence and halt aortic valve calcification progression.

The present study observed that cellular senescence played an essential role in valvular calcification development. Morusin inhibits cellular senescence and osteogenic differentiation of VICs through the Ccnd1/Trim25/Nrf2 signaling pathway. These results highlight morusin's potential therapeutic application in treating CAVD.

## 2 Results

### 2.1 Cellular Senescence was Associated with Valvular Calcification

**Figure 1:**
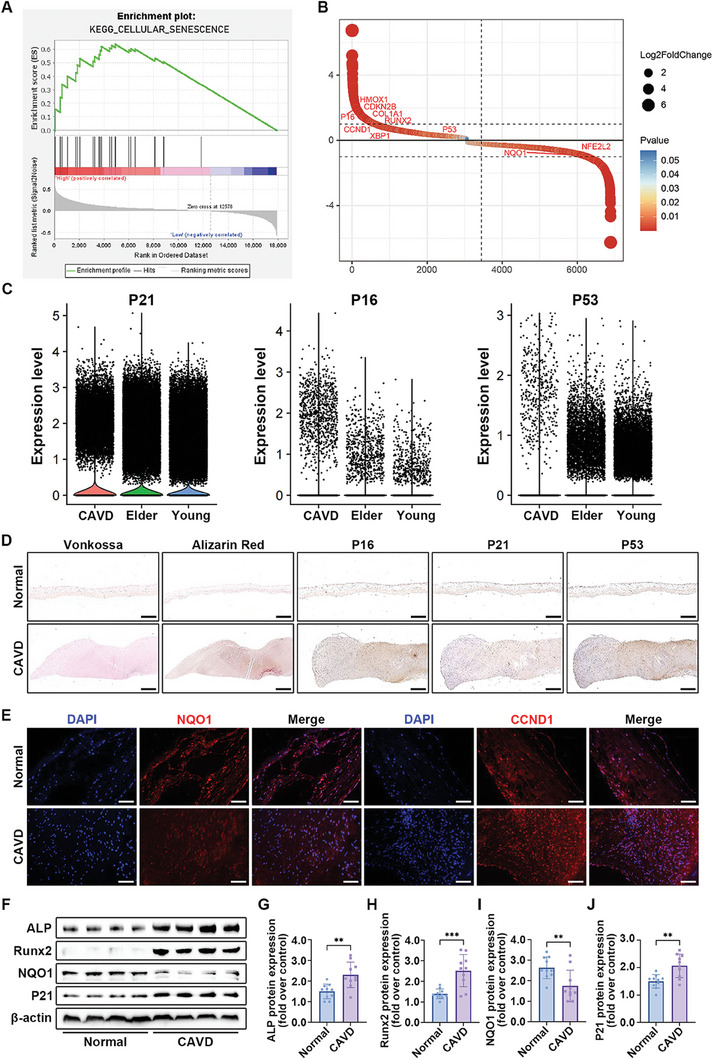
Cellular senescence is associated with aortic valve calcification. A) GSEA plot showing differential expression of signature genes in the cellular senescence pathway in calcific valve and normal valvular tissue based on the RNA‐seq data sets from the GEO database. B) DEG plot showing the differential expression genes between the calcific valve and normal valvular tissue. C) Violin plots representing the expression of P21, P16, and P53 in the aortic valve from CAVD patients, elder and young individuals. D) Representative Vonkossa staining, Alizarin red staining, and immunohistochemical staining of P16, P21, and P53 in aortic valves from CAVD patients and controls. Scale bar 200 µm. E) Immunofluorescent staining of NQO‐1 (red), CCND1 (red), and DAPI (blue) in the aortic valve from CAVD patients and controls. Scale bar 100 µm. F) Protein expression of ALP, Runx2, NQO1, and P21 in the aortic valve from CAVD patients (*n* = 10) and controls (*n* = 10). Bar plots representing the fold change of specific protein expression over control. Data are means ± SD. ^**^ *p* < 0.01; ^***^ *p* < 0.001 (unpaired two‐tailed Student's *t*‐test).

Aortic valve calcification is a degenerative disease related to age and calcification, with studies indicating an association between valvular interstitial cell (VIC) senescence and the progression of this condition.^[^ ^14^ ^]^ RNA sequencing data from aortic tissue obtained from the GEO database revealed through GSEA that cellular senescence positively correlates with valvular calcification (**Figure** **1**A). Cellular senescence entails irreversible cell cycle arrest triggered by stressors such as aging, heightened ROS, or DNA damage.^[^ ^13^ ^]^ Abnormal expression of multiple genes associated with cellular senescence, was observed in calcific aortic tissues. Senescent cells exhibit elevated expression of P16, P21, P53, and CCND1 and downregulated expression of NQO1 and NRF2 (Figure 1B). Single‐cell RNA sequencing analysis of aortic valve tissue further confirmed increased expression of P21, P16, and P53 in calcific aortic valves (Figure 1C). Immunohistochemical assays illustrated the upregulated expression of P16, P21, and P53 in calcific aortic valves, all associated with the cell cycle pathway (Figure 1D). Immunofluorescent staining of the valvular tissue further confirmed increased expression of CCND1 and decreased expression of antioxidant genes, including NQO1, HMXO‐1, and NRF2 in calcific aortic valves (Figure 1E; Figure S1, Supporting Information). Additionally, immunoblot analysis revealed higher expression of ALP, Runx2, and P21, and reduced expression of NQO1 in calcific valvular tissue compared with normal aortic valves (Figure 1E–J). These findings suggest that cellular senescence potentially plays a crucial role in the progression of aortic valve calcification.

### 2.2 Morusin Attenuated OM‐Induced Osteogenic Differentiation of VICs

**Figure 2:**
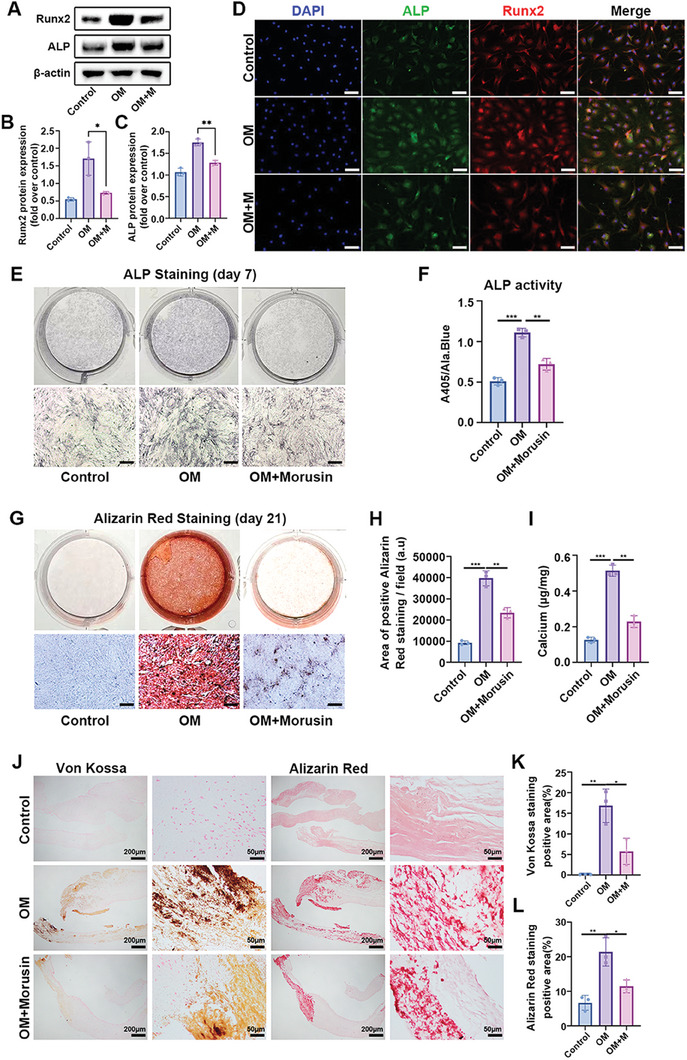
Morusin inhibits OM‐induced osteogenic differentiation of VICs. A) Immunoblot analysis of Runx2 and ALP expression in VICs from indicated groups (*n* = 3, each group). B) Bar plot showing the fold change of Runx2 expression over control. C) Bar plot showing the fold change of ALP expression over control. D) Immunofluorescent staining of ALP (green), Runx2 (red), and DAPI (blue) in the VICs from indicated groups. Scale bar 50 µm. E,F) With OM induction for 7 days, representative ALP staining of VICs from indicated groups (*n* = 3, each group). Scale bar 50 µm. G–I) With OM induction for 21 days, representative Alizarin red staining showed the calcific nodules in VICs from indicated groups (*n* = 3, each group). Scale bar 50 µm. J) With OM induction for 21 days, representative Von Kossa and Alizarin Red staining of aortic valve leaflets. K) Bar plot showing the percentage of Von Kossa positive staining area of indicated groups (*n* = 3, each group). L) Bar plot showing the percentage of Alizarin Red positive staining area of indicated groups (*n* = 3, each group). Data are mean ± SD. ^*^ *p* < 0.05; ^**^ *p* < 0.01; ^***^ *p *< 0.001 (ANOVA with Tukey's multiple comparisons test).

Mulberry extracts exert antioxidant effects, showing the potential to prevent cellular senescence.^[^ ^21^ ^]^ Morusin, isolated from mulberry extracts, has been reported to ameliorate cell senescence.^[^ ^25^ ^]^ To assess the toxicity of morusin on VICs, various concentrations were applied, revealing signs of toxicity at levels exceeding 2 µm (Figure S2A,B, Supporting Information). In the investigation of morusin's therapeutic potential in preventing CAVD, VICs were cultured in OM with or without 1 µm morusin. Under OM induction, VICs upregulated ALP, Runx2, and P21 expression in a time‐dependent manner while downregulating HMXO1 expression (Figure S3, Supporting Information). Immunoblot assays demonstrated that morusin inhibited the OM‐induced expression of osteogenic markers in VICs, including ALP and Runx2 (**Figure** **2**A–C). Subsequently, immunofluorescent staining of ALP and Runx2 on VICs cultured under the same conditions corroborated the results from immunoblot analysis (Figure 2D). After 7 days of OM induction, morusin significantly reduced ALP activity in VICs (Figure 2E,F). Semi‐quantitative analysis using alizarin red staining revealed that morusin notably attenuated OM‐induced calcification of VICs (Figure 2G–I). Furthermore, aortic valve leaflets cultured in OM to induce osteogenic differentiation and treated with morusin displayed reduced calcification, as shown by Von Kossa and alizarin red staining (Figure 2J–L). In vitro experiments strongly suggest that morusin inhibits OM‐induced osteogenic differentiation.

### 2.3 Morusin Modulated the Expression of Senescence‐Related Genes in VICs

**Figure 3:**
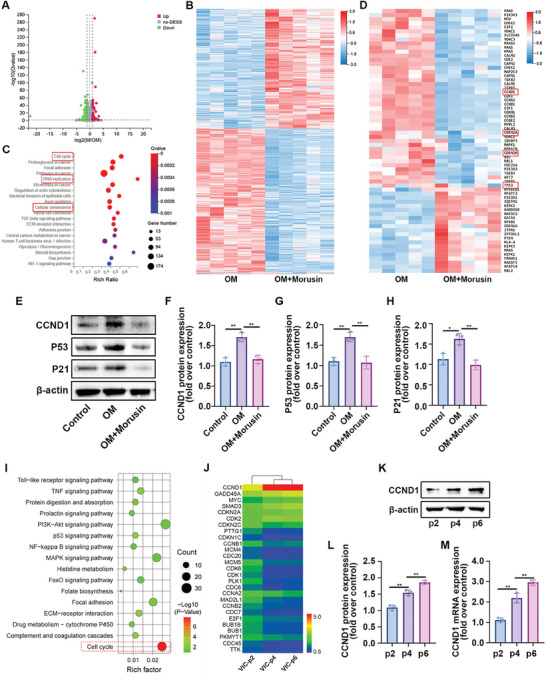
Gene expression profiles of VICs under the OM conditioned culturing with or without morusin. A) Volcano map of differentially expressed genes in OM + morusin versus OM (log_2_M/OM). B) Heatmap for all DEGs in OM + morusin versus OM groups. C) KEGG pathway enrichment of DEGs, bubble colors (deep) indicate the degree of enrichment, bubble size indicates gene counts matched the pathway enrichment, and rich ratio indicates the matched gene counts in the integrated pathway background genes. D) Heatmap for DEGs in cellular senescence pathway. E–H) Immunoblot analysis of CCND1, P53, and P21 expression in VICs from indicated groups (*n* = 3, each group). Bar plots showing the fold change of CCND1, P53, and P21 expression over control. I) KEGG pathway enrichment of DEGs in the second, fourth, and sixth passages of VICs. J) Heatmap for DEGs in cell cycle pathway. K,L) Immunoblot analysis of CCND1 in the second, fourth, and sixth passages of VICs (*n* = 3, each group). Bar plots showing the fold change of CCND1 expression over the second passage of VICs. M) RT‐PCR analysis of CCND1 mRNA level in the second, fourth, and sixth passages of VICs (*n* = 3, each group). Bar plots showing the fold change of CCND1 expression over the second passage of VICs. Data are means ± SD. ^*^ *p* < 0.05; ^**^ *p *< 0.01 (ANOVA with Tukey's multiple comparisons test).

A comparison of gene expression profiles in VICs cultured under OM conditioning, with or without the presence of morusin, was performed. Compared with the group exposed to only OM, VICs in the OM plus morusin group exhibited 3720 DEGs, of which 1687 showed upregulated expression and 2033 showed downregulated expression (**Figure** **3**A). The heatmap displayed all DEGs in VICs between the OM and OM plus morusin groups (Figure 3B). To identify the signaling pathways affected by morusin, KEGG signal pathway enrichment analysis was performed based on these 3720 DEGs. The results highlighted significant enrichment of DEGs in pathways related to the cell cycle, DNA replication, and cellular senescence (Figure 3C). Notably, alterations in cell cycle and DNA replication are common features observed during cellular senescence. The data strongly suggested that morusin mitigates aortic valve calcification through pathways associated with cellular senescence.

Analysis of DEGs associated with cellular senescence from RNA sequencing data indicated that morusin could suppress the expression of CCND1, CDKN2A, CDKN2B, and TP53 in OM‐induced VICs (Figure 3D). A tight association exists between the activation of the CCND1/P53/P21 signaling pathway and VIC calcification.^[^ ^35^ ^]^ In the present investigation, immunoblot analysis demonstrated an increase in the expression of CCND1, P21, and P53 during the osteogenic differentiation of VICs, while treatment with morusin inhibited the CCND1/P53/P21 signals in OM‐induced VICs (Figure 3E–H). Older VIC generations exhibited distinct characteristics of cell senescence. Additionally, mRNA profiles from the second, fourth, and sixth generations of VICs were compared so as to elucidate the dynamic changes in gene expression during VIC senescence. KEGG signal pathway enrichment analysis revealed enrichment of DEGs in the cell cycle pathway (Figure 3I). Particularly, CCND1 expression notably increased in the fourth and sixth generations of VICs compared with the second passage within the cell cycle pathway (Figure 3J). Immunoblot and RT‐PCR assays further validated the elevation of CCND1 with VIC proliferation (Figure 3K–M). These findings underscored the significance of CCND1 in VIC senescence and its susceptibility to inhibition by morusin.

### 2.4 Decreasing CCND1 Inhibited the Osteogenic Differentiation of VICs in Vitro

**Figure 4:**
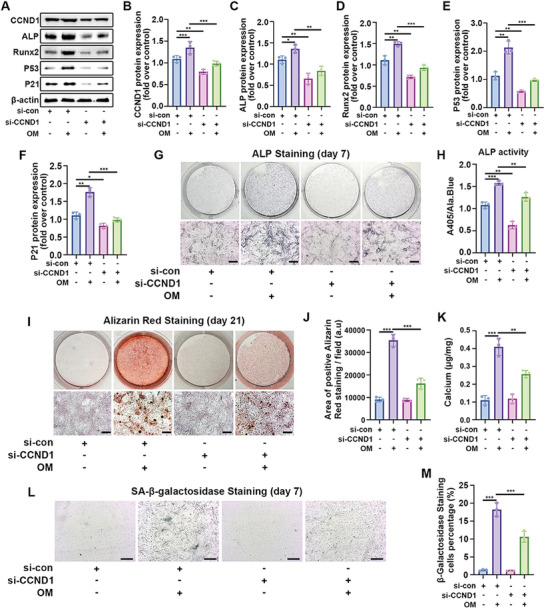
CCND1 participates in the osteogenic differentiation of VICs following OM. A–F) VICs were transfected with CCND1 siRNA or scrambled siRNA, and then stimulated with OM for 7 days. Immunoblot analysis of CCND1, ALP, Runx2 P53, and P21 expression in VICs from indicated groups (*n* = 3, each group). Bar plots showing the semiquantitative analysis of indicated genes expression. G,H) With OM induction for 7 days, representative ALP staining of VICs from indicated groups (*n* = 3, each group). Scale bar 50 µm. I–K) With OM induction for 21 days, representative Alizarin red staining showed the calcific nodules in VICs from indicated groups (*n* = 3, each group). Scale bar 50 µm. L–M) Representative SA‐β‐gal staining of VICs from indicated groups (*n* = 3, each group). Bar plot showing the percentage of SA‐β‐gal staining positive cells. Scale bar 50 µm. Data are means ± SD. ^*^ *p* < 0.05; ^**^ *p* < 0.01; ^***^ *p* < 0.001 (ANOVA with Tukey's multiple comparisons test).

Further investigations were carried out to delineate the role of CCND1 in valvular calcification. VICs were transfected with CCND1 siRNA to suppress CCND1 expression. Three CCND1 siRNAs were designed, synthesized, and evaluated for their knockdown efficiency. Subsequent experiments utilized the third CCND1 siRNA because of its highest efficiency in inhibiting CCND1 expression in VICs (Figure S4, Supporting Information). Diminishing CCND1 expression also led to the inhibition of Collagen I, ALP, P53, and P21 expression in OM‐induced VICs (Figure S4, Supporting Information). Following OM induction, the si‐CCND1 group exhibited significantly lower CCND1 expression than the scrambled siRNA group (**Figure** **4**A). Additionally, decreased CCND1 expression correlated with the suppression of Runx2, ALP, P53, and P21 expression in OM‐induced VICs (Figure 4A–F). Additionally, ALP staining and alizarin red staining indicated that the downregulation of CCND1 mitigated ALP activity and alleviated the degree of calcification in OM‐induced VICs (Figure 4G–K). These findings confirmed the involvement of CCND1 in the progression of valvular calcification. Considering the association between cell senescence and valvular calcification, this study investigated the effect of CCND1 on VIC senescence. Senescent cells can create an aging microenvironment by secreting cytokines, chemokines, and matrix metalloproteinases and can be identified by β‐gal staining.^[^ ^13^ ^]^ The number of β‐gal‐positive VICs significantly increased upon OM stimulation, while CCND1 knockdown reduced the number of OM‐induced β‐gal‐positive VICs (Figure 4L,M).

### 2.5 Morusin Alleviated the Senescence of VICs Through CCND1/Nrf2 Pathway

**Figure 5:**
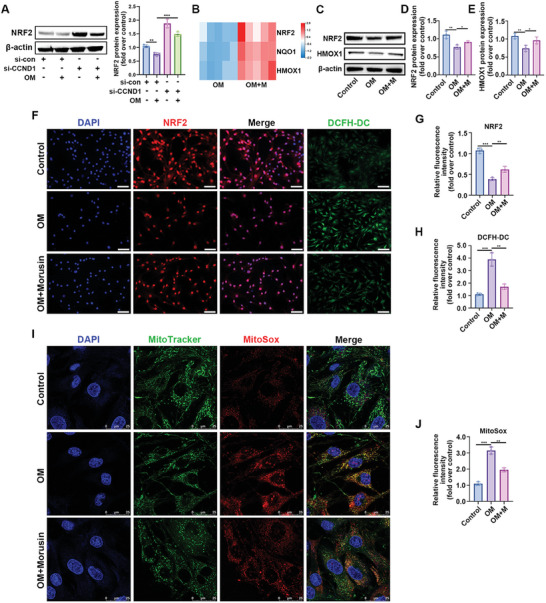
Morusin alleviates the senescence of VICs through CCND1/Nrf2 pathway. A) VICs were transfected with CCND1 siRNA or scrambled siRNA, and then stimulated with OM for 7 days. Immunoblot analysis of Nrf2 expression in VICs from indicated groups (*n* = 3, each group). Bar plots showing the semiquantitative analysis of Nrf2 expression. B) Heatmap for NRF2, NQO1, and HMXO‐1 in OM + morusin versus OM groups. C–E) Immunoblot analysis of NRF2 and HMXO‐1 expression in VICs from indicated groups (*n* = 3, each group). Bar plots showing the fold change of indicated genes expression over control. F–H) Immunofluorescent staining of NRF2 (red), DCFH‐DC (green), and DAPI (blue) in the VICs from indicated groups (*n* = 3, each group). Bar plots showing the semiquantitative analysis of fluorescence intensity. Scale bar 50 µm. I,J) Representative images showing MitoSOX (red) staining and quantification of the fluorescence intensity of MitoSOX fluorescence in VICs from indicated groups (*n* = 3, each group). Data are mean ± SD. ^*^ *p* < 0.05; ^**^ *p* < 0.01; ^***^ *p* < 0.001 (ANOVA with Tukey's multiple comparisons test).

The process of cell senescence often involves oxidative stress and mitochondrial dysfunction.^[^ ^36^ ^]^ Nrf2, a transcription factor, plays a pivotal role in regulating genes responsible for antiaging proteins.^[^ ^37^, ^38^ ^]^ Inhibiting CCND1 significantly increased Nrf2 expression, suggesting a negative regulatory link between CCND1 and Nrf2 (**Figure** **5**A). Subsequently, the effect of morusin on both the expression and function of Nrf2 in VICs was investigated. Predictions indicated morusin's potential interaction with CCND1, validated through the cellular thermal shift assay (Figure S5A,B, Supporting Information). Compared with the OM‐only group, the addition of morusin substantially elevated the expression of NRF2, HMXO‐1, and NQO1 (Figure 5B). Immunoblot and immunofluorescent staining assays confirmed that morusin indeed bolstered Nrf2 expression in OM‐induced VICs (Figure 5C–G). Additionally, morusin increased HMXO‐1 expression in VICs (Figure 5C), known for its antioxidant properties.^[^ ^39^ ^]^ DCFH‐DC staining revealed that morusin effectively inhibited OM‐induced ROS in VICs (Figure 5F,H). Past studies have linked OM to endoplasmic reticulum stress and ROS production in VICs.^[^ ^38^ ^]^ Morusin's effects on ROS generation in OM‐induced VICs demonstrated lower ROS levels than the OM‐only group (Figure 5I,J). These findings collectively suggest that morusin activates the Nrf2‐mediated antiaging effect on VICs.

### 2.6 Nrf2 Inhibitor Reversed the Morusin Mediated Attenuation of VIC Calcification

**Figure 6:**
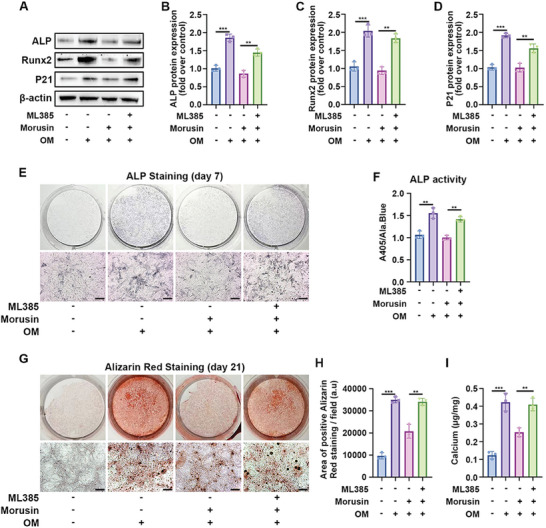
Morusin attenuates VIC calcification by activating Nrf2 signaling pathway. A–D) ML385 was used to inhibit the activation of Nrf2 in VICs. Representative immunoblot images and quantification of the levels of ALP, Runx2, and P21 in VICs from indicated groups (*n* = 3, each group). E,F) With OM induction for 7 days, representative ALP staining of VICs from indicated groups (*n* = 3, each group). Scale bar 50 µm. G–I) With OM induction for 21 days, representative Alizarin red staining of VICs from indicated groups (*n* = 3, each group). Scale bar 50 µm. Data are means ± SD. ^**^ *p* < 0.01; ^***^ *p* < 0.001 (ANOVA with Tukey's multiple comparisons test).

Next, the Nrf2 inhibitor ML385 was utilized to hinder Nrf2 activation. In comparison to the OM plus morusin group, inhibiting Nrf2 increased the expression of Runx2, ALP, p21, NQO1, and HMXO‐1, indicating the reversal of morusin's protective effect on VICs (**Figure** **6**A–D; Figure S6). Furthermore, ALP staining displayed heightened activity in VICs treated with ML385 compared to those in the OM plus morusin group (Figure 6E,F). Additionally, Alizarin red staining indicated morusin's capacity to curb calcific nodule formation in VICs, an effect nullified by ML385 (Figure 6G–I). Overall, these data suggest that morusin functions in an antiaging capacity through Nrf2, impeding the osteogenic differentiation of VICs.

### 2.7 Morusin Increased Trim25 Expression and Keap1 Ubiquitination

**Figure 7:**
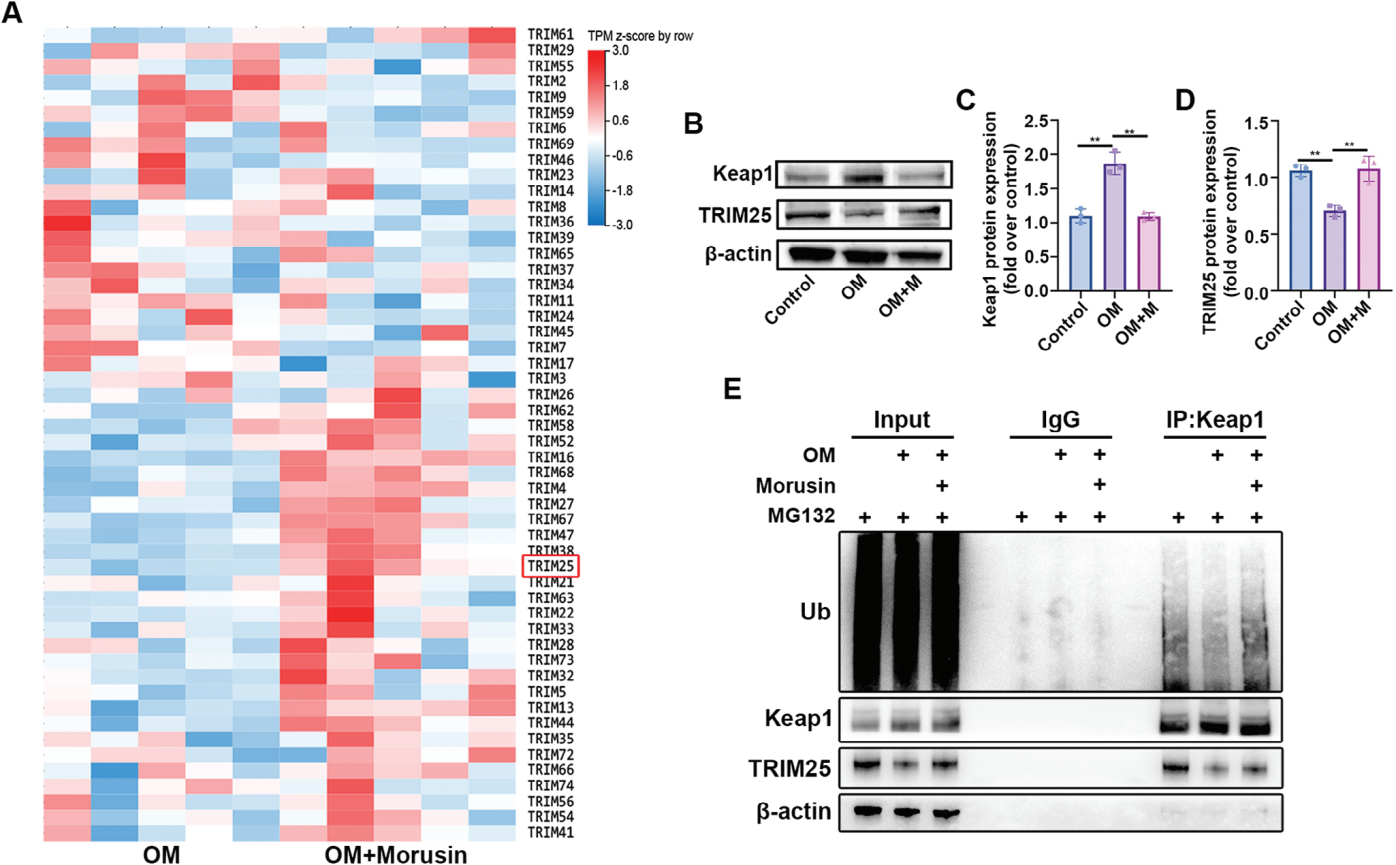
Morusin increases the Trim25 expression and Keap1 ubiquitination level. A) The mRNA expression of TRIM family proteins in OM‐induced VICs treated with or without morusin. B–D) Representative immunoblot images and quantification of the levels of Keap1 and TRIM25 in VICs from indicated groups (*n* = 3, each group). E) Co‐IP analysis of the intercellular combination between Keap1 and TRIM25 and Keap1 ubiquitination level in VICs from indicated groups (*n* = 3, each group). Data are means ± SD. NS, not significant, ^*^ *p* < 0.05, ^**^ *p* < 0.01, ^***^ *p* < 0.001 (ANOVA with Tukey's multiple comparisons test.

Keap1 acts as a negative regulator of Nrf2 by binding to it, hindering Nrf2 from entering the nucleus.^[^ ^40^ ^]^ Keap1 degradation enables Nrf2 translocation to the nucleus, thereby enhancing downstream protein expression.^[^ ^40^, ^41^ ^]^ The Trim family of E3 ligases regulates Keap1 homeostasis through ubiquitination, with Trim25 being identified to interact with and promote Keap1 ubiquitination.^[^ ^42^ ^]^ In this study, morusin's effect on Trim family expression was explored, revealing an increase in Trim proteins, specifically Trim16 and Trim25, in OM‐induced VICs (**Figure** **7**A). Nevertheless, knockdown experiments targeting Trim16 indicated minimal influence on VIC calcification (Figure S7, Supporting Information). Protein analysis of Keap1 and Trim25 in VICs during osteogenic differentiation displayed increased Keap1 expression and reduced Trim25 expression (Figure 7B). Morusin administration reduced Keap1 levels and augmented Trim25 expression in OM‐induced VICs (Figure 7B–D). Co‐IP detection demonstrated that morusin heightened Trim25 expression and Keap1 ubiquitination levels in OM‐induced VICs (Figure 7E). Consequently, morusin increased Trim25‐mediated ubiquitination degradation of Keap1, fostering Keap1 degradation to facilitate Nrf2 translocation and activation.

### 2.8 Morusin Alleviated the Calcification of VICs Through CCND1/Trim25 Pathway

**Figure 8:**
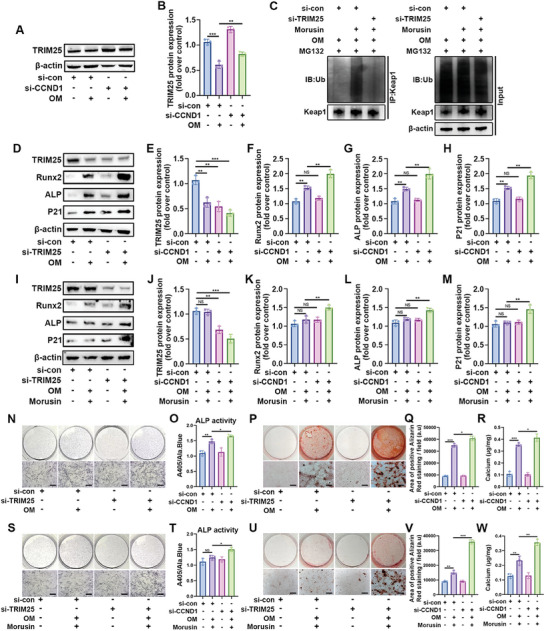
Morusin attenuates VIC calcification depending on Trim25. A,B) VICs were transfected with CCND1 siRNA or scrambled siRNA, immunoblot analysis of Trim25 expression in VICs from indicated groups (*n* = 3, each group). Bar plots showing the semiquantitative analysis of Trim25 expression. C) VICs were transfected with Trim25 siRNA or scrambled siRNA. Co‐IP analysis of the Keap1 ubiquitination level in VICs from indicated groups (*n* = 3, each group). D–M) Representative immunoblot images and quantification of the levels of Trim25, ALP, Runx2, and P21 in VICs from indicated groups (*n* = 3, each group). N,S) VICs were transfected with Trim25 siRNA or scrambled siRNA, representative ALP staining of VICs from indicated groups (*n* = 3, each group). P,U) VICs were transfected with Trim25 siRNA or scrambled siRNA, representative Alizarin red staining of VICs from indicated groups (*n* = 3, each group). Scale bar 50 µm. Data are means ± SD. NS, not significant, ^*^ *p* < 0.05, ^**^ *p* < 0.01, ^***^ *p* < 0.001 (ANOVA with Tukey's multiple comparisons test).

Subsequently, this study investigated whether CCND1 influenced the protein levels of Nrf2 in a Trim25‐dependent manner. Upon OM induction, CCND1 knockdown significantly increased the protein level of Trim25 (**Figure** **8**A,B). In OM‐induced VICs, administration of morusin increased the ubiquitination level of Keap1. However, such an effect could be abolished by Trim25 knockdown, indicating that the ubiquitination of Keap1 was mediated through Trim25 (Figure 8C). During the osteogenic differentiation of VICs, Trim25 expression was downregulated, while the levels of P21, ALP, and Runx2 were elevated (Figure 8D). Knockdown of Trim25 further increased the expression of P21, ALP, and Runx2 in OM‐induced VICs (Figure 8D–H). Morusin administration increased Trim25 levels in OM‐induced VICs, and Trim25 knockdown abolished morusin‐mediated downregulation of P21, ALP, and Runx2 expression (Figure 8I–M). Moreover, decreased Trim25 further increased ALP activity in OM‐induced VICs (Figure 8N,O). Morusin inhibited ALP activity in OM‐induced VICs, an effect inhibited by Trim25 knockdown (Figure 8S,T). Additionally, alizarin red staining was performed to investigate whether Trim25 influenced the formation of calcific nodules in VICs. The result showed more calcific nodules in OM‐induced VICs transfected with si‐Trim25 (Figure 8P–R). Trim25 knockdown interfered with morusin‐mediated attenuation of VIC calcification (Figure 8U–W). Based on these results, Trim25 was negatively regulated by CCND1, and morusin inhibited the osteogenic differentiation of VICs in a Trim25‐dependent manner.

### 2.9 Morusin Alleviated Aortic Valve Calcification in ApoE^−/−^ Mice

**Figure 9:**
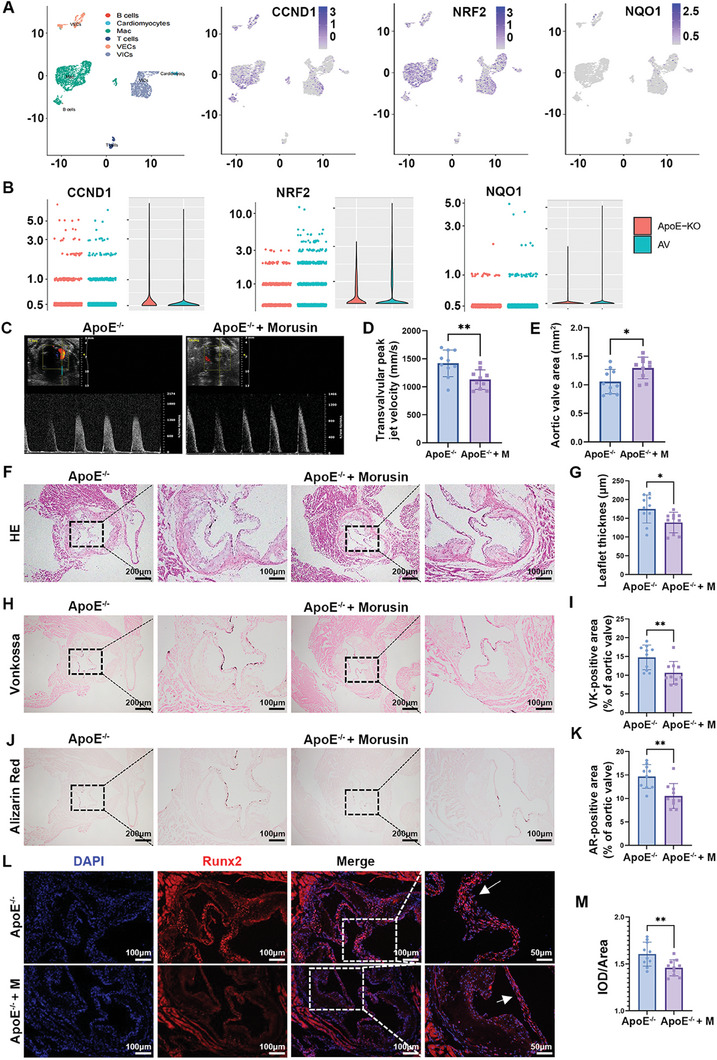
Morusin prevents the aortic valve calcification in ApoE^−/−^ mice. A) UMAP plot of cells from mouse aortic valve tissue, color‐coded by expression of marker genes. B) Violin plots representing the expression of CCND1, NRF2, and NQO1 in the VICs from mouse aortic valve tissue. C,D) Echocardiographic assessment showing morusin alleviates the aortic valve dysfunction (peak transvalvular velocity) induced by WD (*n* = 10, each group). E,F) Representative H&E stain images and quantitative analysis showing morusin alleviates aortic valve thickening induced by WD (*n* = 10, each group). G–H) Representative Vonkossa staining of mouse aortic valve from indicated groups (*n* = 10, each group). Bar plot showing the percentage of VK positive area. I,J) Representative Alizarin red staining of mouse aortic valve from indicated groups (*n* = 10, each group). Bar plot showing the percentage of AR‐positive area. K,L) Immunofluorescent staining of Runx2 (red) and DAPI (blue) in the mouse valvular tissue from indicated groups (*n* = 10, each group). Bar plot showing the semiquantitative analysis of fluorescence intensity. Data are means ± SD. ^*^ *p *< 0.05; ^**^ *p *< 0.01 (unpaired two‐tailed Student's *t*‐test).

**Figure 10:**
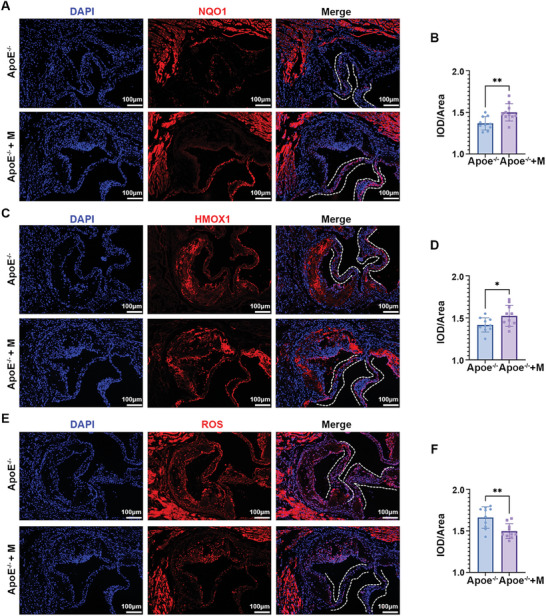
Morusin prevents the valvular calcification via its antioxidant effects in vivo. A,B) Immunofluorescent staining of NQO1 (red) and DAPI (blue) in the mouse valvular tissue from indicated groups (*n* = 10, each group). Bar plot showing the semiquantitative analysis of fluorescence intensity. C,D) Immunofluorescent staining of HMXO‐1 (red) and DAPI (blue) in the mouse valvular tissue from indicated groups (*n* = 10, each group). Bar plot showing the semiquantitative analysis of fluorescence intensity. E,F) Immunofluorescent staining of ROS (red) and DAPI (blue) in the mouse valvular tissue from indicated groups (*n* = 10, each group). Bar plot showing the semiquantitative analysis of fluorescence intensity. Bar plot showing the semiquantitative analyzes of fluorescence intensity. Data are mean ± SD. ^*^ *p *< 0.05; ^**^ *p *< 0.01 (unpaired two‐tailed Student's *t*‐test).

To investigate the therapeutic effect of morusin in vivo, single‐cell RNA‐seq data of mouse aortic valve tissue from the GEO database (GSE180278) were analyzed.^[^ ^43^ ^]^ Ccnd1, NRF2, and Nqo1 were commonly expressed in VEC, VIC, and macrophages (**Figure** **9**A; Figure S8A–C, Supporting Information). Inflammatory activation was observed in calcific valves (Figure S9, Supporting Information). Additionally, compared with control mice, ApoE^−/−^ mice showed increased Ccnd1 expression and decreased Nrf2 and Nqo1 expression in VICs, consistent with the aforementioned results (Figure 9B). Subsequently, ApoE^−/−^ mice were fed a high‐fat western diet for an extended period to establish an aortic valve calcification model. The comparison of echocardiographic and hemodynamic parameters in ApoE^−/−^ mice is presented in Table S4 (Supporting Information). To assess the therapeutic effect of morusin on CAVD, serum lipids, thickness, calcification, function, aortic valve area, and osteogenesis gene expression in valvular tissue were evaluated of ApoE^−/−^ mice fed a western diet with or without morusin treatment. Morusin had little influence on the serum lipids in ApoE^−/−^ mice (Figure S10, Supporting Information). The echocardiographic assessment revealed that morusin significantly decreased aortic valve peak velocity and increased the aortic valve area in ApoE^−/−^ mice fed with a western diet (Figure 9C–E). Histological analysis indicated a significant reduction in aortic valve thickening in ApoE^−/−^ mice treated with morusin (Figure 9F,G). Furthermore, Von Kossa and alizarin red staining showed that morusin inhibited microcalcification of the aortic valve in ApoE^−/−^ mice (Figure 9H–K). Immunofluorescent staining demonstrated that morusin inhibited the expression of Runx2 in the valvular tissue (Figure 9L,M). Additionally, morusin reduced ROS levels and increased the expression of antioxidant genes, including NQO1 and HMXO‐1, in the aortic valve of ApoE^−/−^ mice (**Figure** **10**A–F). This study examined the expression of senescence‐associated genes in the valvular tissue and found that morusin significantly reduced the expression of P16, P21, P53, and CCND1 (Figure S11, Supporting Information) while increasing the expression of TRIM25 and NRF2 (Figure S12, Supporting Information) in the valvular tissue of ApoE^−/−^ mice. ALP staining of the mouse aortic valve revealed that morusin reduced ALP levels (Figure S13, Supporting Information). In conclusion, these results suggest that morusin has great potential to attenuate aortic valve calcification.

## 3 Discussion

**Figure 11:**
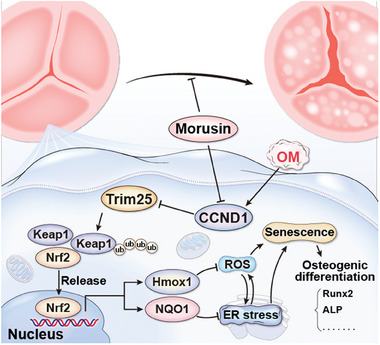
Diagram of morusin‐mediated protective effect on CAVD progression.

With increasing life expectancy, CAVD has emerged as the most prevalent cardiovascular ailment affecting the heart health of elderly individuals. The pathogenesis of CAVD is intricate, involving reported contributions from inflammation, cellular senescence, and osteogenic differentiation in its progression.^[^ ^44^ ^]^ Thus, it is imperative to elucidate the molecular mechanisms driving the onset of CAVD and to develop novel drugs aimed at preventing its progression. This study observed therapeutic properties of morusin, a natural compound isolated from mulberry, in averting aortic valve calcification. VIC senescence plays a pivotal role in CAVD progression, with multiple senescent genes showing abnormal expression in calcified valves. Morusin exhibited an anti‐aging effect by ameliorating VIC senescence and inhibiting valvular calcification. Mechanistically, morusin reduced the expression of CCND1. The consequent downregulation of CCDN1 increased Trim25‐mediated ubiquitination degradation of Keap1, subsequently promoting Nrf2‐mediated expression of antiaging genes (**Figure** **11**). Several clinical trials have demonstrated the therapeutic effects of Mulberry leaf extracts on cardiovascular diseases and diabetes.^[^ ^45^, ^46^, ^47^ ^]^ Collectively, the data suggest that morusin holds significant potential for treating CAVD, warranting clinical trials to evaluate its safety and efficacy in practical application. The in vivo process of aortic valve calcification is intricate and highly dynamic. To investigate the mechanism of valvular calcification, OM is commonly used for in vitro experiments. Under OM stimulation, normally fibroblastic VICs undergo osteogenic differentiation, characterized by upregulated osteoblastic markers and the formation of calcific nodules. In this study, VICs exhibited increased ALP and Runx2 expression with more prominent calcific nodule formation under OM stimulation. Although OM cannot perfectly replicate the complex in vivo environment, it remains an essential method for assessing the effectiveness of morusin in treating aortic valve calcification.

Cellular senescence is reportedly linked to various age‐related chronic diseases, including CAVD.^[^ ^48^, ^49^, ^50^ ^]^ One of the defining characteristics of cellular senescence is irreversible cell cycle arrest. Senescent cells are typically arrested in the G1/S or G2 phase, losing their ability to proliferate.^[^ ^51^ ^]^ Several genes related to the cell cycle have shown abnormal expression in senescent cells. For instance, senescent cells may upregulate the expression of p53, p21, and p16.^[^ ^52^, ^53^, ^54^ ^]^ The activation of the p53/p21 pathway is associated with cell cycle arrest at G1/S or G2/M, while the combination of p16 and CDK4/6 can prevent Rb phosphorylation, leading to G1/S cell cycle arrest. Cyclin D1 (CCND1), a key regulator of the cell cycle, is known to promote osteogenic differentiation.^[^ ^55^ ^]^ This study observed high expression levels of CCND1, p53, p21, and p16 in calcific valves and osteogenic VICs, indicating the involvement of the cell cycle pathway in valvular calcification. Activation of the CCND1/P53/P21 signal could inhibit cell proliferation and promote cell differentiation.^[^ ^35^ ^]^ The results demonstrate a positive correlation between the protein levels of CCND1, P53, and P21 with the expression of osteogenic markers in VICs. Additionally, this study demonstrated a significant increase in CCND1 expression in aging VICs, underscoring its critical role in cellular senescence. Furthermore, the administration of morusin suppressed the CCND1/P53/P21 signal and osteogenic differentiation of VICs, suggesting the antiaging effect of morusin in mitigating aortic valve calcification.

Moreover, the findings revealed a negative correlation between the protein level of CCND1 and the expression of Nrf2. Using Si‐CCND1 to reduce CCND1 expression in VICs resulted in the inhibition of OM‐induced cellular senescence and osteogenic differentiation. CCND1 knockdown increased Nrf2 expression levels in VICs, suggesting the regulatory role of CCND1 in Nrf2 expression. However, further investigation is required to elucidate the specific CCND1‐mediated regulatory mechanisms of Nrf2. Several studies have highlighted Nrf2 as a crucial intracellular antioxidant transcription factor.^[^ ^56^ ^]^ In the present study, Nrf2 expression was lower in the calcific valvular tissue. RNA sequencing data indicated that morusin increased Nrf2 expression. Additionally, intervention with the Nrf2 inhibitor ML385 reversed the protective effects of morusin. These results suggest that morusin prevents VIC senescence through the Nrf2‐mediated pathway. Single‐cell RNA sequencing data shown in Figure 9 suggest the expression of common genes such as CCND1 and NRF2 in VECs, VICs, and macrophages. Inflammation is a significant inducer of valvular calcification, and Nrf2 is associated with macrophage inflammatory response. Human calcific valves exhibited more severe inflammation than normal valves (Figure S9, Supporting Information). Morusin also exhibited anti‐inflammatory properties, and further studies are needed to explore its role in regulating immune cell response in aortic valves.

While the mechanisms of cellular senescence are complex, recent studies have confirmed mitochondrial dysfunction and an imbalanced redox system in senescent cells.^[^ ^57^, ^58^, ^59^ ^]^ Combi et al. reported altered mitochondrial metabolism leading to aortic valve calcification.^[^ ^60^ ^]^ Zhang et al. observed oxidative stress participating in alveolar epithelial cell senescence, contributing to pulmonary fibrosis.^[^ ^42^ ^]^ In this study, osteogenic VICs showed increased ROS production in mitochondria. Nrf2 translocation to the nucleus can activate the expression of NQO1 and HMXO‐1. NQO1, a ubiquitous flavoenzyme that catalyzes quinones, utilizes NADH, and generates NAD^+^, plays a crucial role in maintaining intercellular homeostasis and protecting DNA from oxidative stress.^[^ ^61^, ^62^ ^]^ Heme oxygenase‐1 (HMXO‐1), a well‐known antioxidant, is found to alleviate ER stress.^[^ ^63^, ^64^ ^]^ The data indicated downregulated expression of NQO1 and HMXO‐1 in calcific valvular tissue and OM‐induced VICs, indicating an antioxidant deficiency leading to valvular calcification. Furthermore, morusin administration increased the protein levels of NQO1 and HMXO‐1 in OM‐induced VICs, suggesting its potential in restoring the antioxidant properties of these VICs.

Keap1 is a negative regulator of Nrf2.^[^ ^40^ ^]^ The combination of Keap1 and Nrf2 impedes the nuclear translocation of Nrf2, thereby inhibiting the transcription of antioxidant genes.^[^ ^65^ ^]^ Keap1 undergoes degradation through ubiquitination, with the Trim family of E3 ligases being involved in Keap1 degradation.^[^ ^66^, ^67^ ^]^ Zhang et al. discovered that Trim25 promotes the ubiquitination of Keap1 in alveolar epithelial cells, and reducing Trim25 expression inhibited the degradation of Keap1.^[^ ^42^ ^]^ Upon analyzing the RNA sequencing data, a significant increase in Trim25 expression was observed following treatment with morusin. Extracting proteins from VICs for Co‐IP, the results indicated that morusin promotes Trim25‐mediated ubiquitination degradation of Keap1. Additionally, using siRNA to inhibit CCND1 or Trim25 expression, the data revealed CCND1's negative regulation of Trim25 protein levels. Furthermore, knocking down Trim25 nullified the protective effects of morusin, emphasizing the role of Trim25 in morusin‐mediated alleviation of VIC calcification. In conclusion, morusin can activate the Nrf2‐mediated antiaging effect on VICs through the CCND1/Trim25/Nrf2 pathway.

The in vitro experiments in this study demonstrated that morusin possesses antioxidant properties associated with attenuating VIC senescence and improving VIC osteogenic differentiation. Subsequently, this study investigated morusin's therapeutic effect in vivo. Prolonged consumption of a western diet by ApoE^−/−^ mice leads to aortic valve calcification. In the present study, morusin notably alleviated western diet‐induced valvular calcification in ApoE^−/−^ mice, accompanied by significantly higher expression of NQO1 and HMXO‐1 in the morusin treatment group. Moreover, morusin administration reduced ROS production and the expression of genes associated with cellular senescence in mouse aortic valves. These findings suggest that morusin holds promise in preventing the progression of valvular calcification in vivo.

However, this study has certain limitations. First, morusin exhibits anti‐inflammatory properties, and chronic inflammation contributes to the pathogenesis of CAVD.^[^ ^8^ ^]^ However, this study did not provide data on how morusin affects the immune microenvironment of aortic valve tissue. Second, in the presence or absence of morusin, 3720 DEGs in VICs were identified, indicating that other signaling pathways might be modulated by morusin. Further analysis of RNA sequencing data is required. Nonetheless, this study provides evidence supporting the potential of morusin in preventing the progression of valvular calcification. More clinical studies are warranted to investigate its safety and efficacy in CAVD treatment.

## 4 Experimental Section

### Patient Recruitment and Sample Collection

Calcified aortic valves were procured from patients who had CAVD and were undergoing aortic valve replacement at the Department of Cardiovascular Surgery at Wuhan Union Hospital. Patients with rheumatic disease, infective endocarditis, bicuspid aortic valves, or any other significant valvular disease were excluded from the study. Noncalcified aortic valves, confirmed through cardiac ultrasonic examination, were obtained from patients who had undergone heart transplantation. Table S1 (Supporting Information) presents the demographic and baseline clinical characteristics of patients with CAVD and control subjects. All participants provided informed consent. This study was conducted in accordance with the Declaration of Helsinki and approved by the Ethics Committee (No: UHCT‐IEC‐SOP‐016‐03‐01) of Union Hospital and Tongji Medical College.

### Reagents and Antibodies

Chemicals used in the study were procured from different sources: morusin (S0915) from Selleck Chemicals, DAPI (D8417) and dimethyl sulfoxide (C6164) from Sigma Aldrich, Nrf2 inhibitor ML385 (S8790) from Proteintech, Von Kossa staining kit from Sigma–Aldrich, Oil Red O staining kit (G1015) from Sigma–Aldrich, and senescence‐associated β‐galactosidase (SA‐β‐gal) staining kit from Beyotime. Additionally, various assay kits such as triglyceride assay kit (A110‐1‐1), total cholesterol assay kit (A111‐1‐1), high‐density lipoprotein cholesterol assay kit (A112‐1‐1), and low‐density lipoprotein cholesterol assay kit (A113‐1‐1) were procured from Nanjing Jiancheng Bioengineering Institute. The antibodies used for immunoblot, immunofluorescence, and immunohistochemical staining assays were as follows: anti‐actin (81115‐1‐RR, Proteintech), anti‐P21 (2947S, CST), anti‐RUNX2 (8486, CST), anti‐ALP (MAB29092, R&D), anti‐TRIM25 (67314‐1‐Ig, Proteintech), anti‐TRIM16 (24403‐1‐AP, Proteintech), anti‐KEAP1 (10503‐2‐AP, Proteintech), anti‐NQO1 (DF6437, Affinity), anti‐HMXO‐1 (DF8020, Affinity), anti‐NRF2 (AF0639, Affinity), anti‐Cyclin D1 (DF6386, Affinity), anti‐P53 (AF0865, Affinity), anti‐P16 (AF5484, Affinity), anti‐IL6 (21865‐1‐AP, Proteintech), anti‐NF‐κB (66535‐1‐Ig, Proteintech), and anti‐collagen I (ab138492, Abcam).

### Animal Model and Treatment

Eight‐week‐old male ApoE^−/−^ mice were purchased from GemPharmatech (Nanjing, China) and housed in a specific pathogen‐free facility. The mice were randomly divided into two groups and subjected to different dietary conditions. A high‐cholesterol diet (Changzhou SYSE Bio‐tech. Co., Ltd.) was administered to both groups. The first group received a high‐fat Western diet (0.15% cholesterol and 40% calories from fat) for 24 weeks to induce aortic valve calcification. The second group fed the same diet but was additionally treated with morusin twice a week. Morusin was dissolved in physiological saline. Based on the body weight of each mouse, 40 mg k^−1^g of morusin was administered to the mice by gavage. At the end of the treatment period, Doppler ultrasound and M‐mode echocardiography were performed using high‐resolution ultrasound (18–38 MHz) to evaluate the aortic valve function. Subsequently, the mice were euthanized by administering an intravenous injection of a lethal dose of pentobarbital sodium (100 mg k^−1^g), and their aortic valve samples were collected for further analyses, including immunofluorescence and Von Kossa staining assays. Blood samples were collected from the retro‐orbital sinus of mice and transferred to 1.5 mL new Eppendorf tubes. The blood was allowed to clot for 30–60 min at 37 ˚C, and the serum was centrifuged at 10 000 × *g* for 10 min at 4 ˚C to remove any residual insoluble material, and the serum was transferred to a new tube for serum lipid detection. Animal studies were conducted after obtaining ethical approval ([2022] IACUC No: 3493) from the Animal Care and Use Committee of Union Hospital, Tongji Medical College, Huazhong University of Science and Technology.

### Histology

Aortic valves were fixed with formalin, embedded in paraffin, and sectioned into 5 µm blocks. These blocks were then dewaxed, rehydrated, and stained with hematoxylin‐eosin following the manufacturer's instructions. The tissue sections were examined using a light microscope for evaluation.

### Measurements of Serum Lipids

Blood samples were collected from the retro‐orbital sinus of mice and transferred to 1.5 mL new Eppendorf tubes. The blood was allowed to clot for 30–60 min at 37 °C. Subsequently, the serum was centrifuged at 10 000 × *g* for 10 min at 4 °C to eliminate any residual insoluble material, and the serum was transferred to a new tube. Serum triglycerides, total cholesterol, high‐density lipoprotein cholesterol, and low‐density lipoprotein cholesterol levels were measured using the triglyceride assay kit, total cholesterol assay kit, high‐density lipoprotein cholesterol assay kit, and low‐density lipoprotein cholesterol assay kit, respectively, according to the manufacturer's instructions.

### Isolation and Culture of Human VICs

Human VICs were acquired from noncalcified aortic valve specimens. After washing the aortic valves three times in phosphate‐buffered saline (PBS), they were cut into small pieces and digested in 1 mg mL type I collagenase for 12 h at 37 °C. Subsequently, the undigested tissue was removed using a 70 µm nylon cell filter, and the isolated cells were cultured in high glucose‐Dulbecco's Modified Eagle Medium (DMEM) supplemented with 10% fetal bovine serum and 1% penicillin–streptomycin at 37 °C in an atmosphere with 5% CO_2_. Cells from the third passage were utilized for subsequent experiments. In certain experiments, cells from different passages were used, and this information was specified in the figure legend.

### Ex Vivo Osteogenic Differentiation

VICs were cultured in 12‐well plates at a specific density per well. Osteogenic differentiation of VICs was induced in osteogenic medium (OM) comprising DMEM supplemented with 2% fetal bovine serum, 1% penicillin–streptomycin, 10 mmol L^−1^ of β‐glycerophosphate, 0.1 µmol L^−1^ of dexamethasone, and 50 µg mL^−1^ ascorbic acid. The OM was replenished every 2–3 days. The treatment groups were as follows: control (without OM and morusin), OM alone, and OM plus morusin (with OM and 1 µm morusin). In some experiments, VICs were treated with ML385.^[^ ^68^ ^]^ Human aortic valve leaflets were obtained from patients undergoing heart transplantation. These samples were cut into small 1 mm ×  1 mm pieces, and osteogenic differentiation was induced in OM for 30 days.

### Cell Viability Assay

The cells were cultured in a 48‐well plate at a density of 1 × 10^4^ cells per well. The medium was then replaced with a low‐serum medium to induce starvation of the cells for 12 h, followed by treatment with various concentrations of morusin (0–5 µm) for 72 h. Cell viability was measured using the Calcein/PI Cell Viability/Cytotoxicity Assay Kit (Beyotime) and the CCK‐8 assay (Beyotime) according to the manufacturer's instructions.

### Alkaline Phosphatase Staining

VICs were harvested, washed thrice in PBS, and fixed in 4% paraformaldehyde. Subsequently, the cells were incubated with the BCIP/NBT substrate solution at room temperature in the dark for 15 min. After washing off the substrate solution, images were captured using a light microscope (Mshot), and ALP‐positive cells appeared as a dark blue‐violet color. Image‐Pro Plus software was employed to analyze the positively stained area according to the method used in a previous study.^[^ ^11^, ^69^ ^]^

### SA‐β‐gal Staining

VICs subjected to different treatments were cultured in six‐well plates. Following three washes in PBS, VICs were fixed for 15 min and then incubated with the SA‐β‐gal staining solution at 37 °C overnight. SA‐β‐gal‐positive cells were randomly imaged using a light microscope (Mshot). Image‐Pro Plus software was used to quantify the percentage of cells with positive staining.

### Immunohistochemical Staining

Aortic valves were fixed in 4% paraformaldehyde and embedded in paraffin. The paraffin‐embedded valvular samples were sectioned into 5 µm‐thick sections. The sections of valvular tissue were then deparaffinized, rehydrated, underwent antigen retrieval and endogenous peroxidase inactivation, followed by blocking with 5% bovine serum albumin (BSA). The sections were incubated overnight at 4 °C with primary antibodies. Subsequently, peroxidase‐labeled secondary antibodies were used for 30 min at room temperature, and DAB was utilized as a substrate for staining.

### Immunofluorescence Staining

Frozen sections of valvular tissue were deparaffinized, and endogenous peroxidase was blocked with 3% H_2_O_2_. VICs were collected, washed thrice in PBS, fixed in 4% paraformaldehyde, and permeabilized with 0.1% Triton X‐100 in PBS for 15 min. After two washes in PBS, cells were blocked in 5% BSA for 30 min and then incubated with primary antibodies at 4 °C overnight. Subsequently, the tissue sections were incubated with fluorescence‐labeled secondary antibodies at room temperature for 30 min in the dark. After two PBS washes, DAPI staining was performed at room temperature for 15 min in the dark. Images were captured using a fluorescence microscope, and semi‐quantitative data were presented as integral optical density per area (Nikon, Japan).

### Calcification Analysis

The aortic valvular tissue sections were deparaffinized for calcification analysis. After 21 days of osteogenic differentiation, VICs were washed twice in PBS, fixed in 4% paraformaldehyde, and evaluated for calcium deposition. Samples were stained with alizarin red and Von Kossa according to the manufacturer's instructions. Briefly, VIC monolayers were washed twice with PBS and fixed for 15 min in 4% paraformaldehyde, followed by staining with 0.2% alizarin red solution (pH 4.0–4.2) for 30 min, followed by washing to remove excess dye. To quantify calcium deposition, stained cells were treated with 10% acetic acid at 75 °C, and the supernatant was analyzed spectrophotometrically at 450 nm. Images were captured using an optical microscope (Olympus, Japan), and Image‐Pro Plus software was utilized to evaluate calcium deposits.

### Mitochondrial ROS Measurements

VICs were initially collected and washed thrice in PBS before fixation in 4% paraformaldehyde. After fixation, the cells were washed twice in PBS and subsequent permeabilization using 0.1% Triton X‐100 for 15 min. Mitochondrial ROS were detected using the fluorescent probe MitoSOX (Invitrogen) according to the manufacturer's instructions. Additionally, mitochondria were labeled with MitoTracker Green (Beyotime, Shanghai) according to the manufacturer's instructions.

### Immunoblot Analysis

Proteins extracted from the aortic valve or VICs were lysed in radioimmunoprecipitation assay lysis and extraction buffer (Beyotime, China) containing protease and phosphatase inhibitor cocktail (Thermo Fisher Scientific, USA) on ice for 10 min. After centrifugation at 12 000 × *g* for 10 min at 4 °C, the resulting supernatants were transferred to new Eppendorf tubes. The expression of the target protein was determined using specific antibodies following standard procedures.

### Co‐Immunoprecipitation Assay

VICs, cultivated in 100 mm dishes, were treated with MG132, OM, and morusin. Post‐treatment, the medium was removed, and cells were washed with PBS. Subsequently, cell lysis was performed using 1 mL of cell lysis buffer for western blotting and immunoprecipitation (IP) without inhibitors (P70100, NCM Biotech). After collecting the lysate by centrifugation at 15 000 × *g* for 15 min at 4 °C, a pre‐cleaning step was performed by adding protein A beads into the lysate and incubating for 1 h at 4 °C, followed by another centrifugation at 15 000 × *g* for 15 min at 4 °C. Immunoprecipitation of the supernatants was conducted by incubating with anti‐Keap1 antibody overnight at 4 °C, followed by the addition of protein A agarose beads suspension to the mixture and further incubation for 4 h at 4 °C. The resulting precipitates were washed thrice using cold IP lysis buffer. After the final wash, the beads were resuspended in the sample buffer and denatured at 95 °C for 10 min. The immunoprecipitated proteins or whole‐cell lysates were then analyzed using western blotting using the antibodies specified in the figures.

### RNA Isolation and RNA‐Sequencing Analysis

Total RNA from VICs was isolated using the Direct‐zol RNA MicroPrep Kit (Zymo Research, USA) following the manufacturer's instructions. RNA quality was assessed using NanoDrop (Thermo Fisher Scientific, USA). Samples were then subjected to RNA‐sequencing by BGI Co., Ltd. (Shenzhen, China), using a BGISEQ‐500 instrument. The obtained sequencing data were further analyzed to identify differentially expressed genes (DEGs), and Kyoto Encyclopedia of Genes and Genomes (KEGG) pathway enrichment analysis was performed using R Language.

### Cellular Thermal Shift Assay

VICs were treated with or without morusin for 1 h. Subsequently, 100 µL of each cell suspension was aliquoted into six polymerase chain reaction (PCR) tubes and heated at different temperatures for 2 min using an Applied Biosystems PCR analyzer.^[^ ^70^ ^]^ The supernatant, obtained through centrifugation, was analyzed using western blotting analysis.

### Dataset Acquisition and Processing

The expression profiles of aortic valve tissues (GSE138531, GSE148219, GSE153555, GSE55492, and GSE76717) were downloaded from the European Nucleotide Archive (ENA) repository for subsequent analysis. Sequencing data were analyzed to identify DEGs. The limma package in R was utilized to identify DEGs between OM samples and OM plus morusin samples. Genes with a *p* value of <0.05 and |logFC| > 1 were considered as DEGs in this dataset. Gene set enrichment analysis (GSEA) was performed using R Language. Additionally, single‐cell RNA sequencing data of mouse aortic valvular tissue (GSE180278) were obtained from the Gene Expression Omnibus (GEO) database and analyzed using the R package Seurat (v3.1.1). Human scRNA‐seq data were sourced from the Sequence Read Archive database of NCBI under the accession number PRJNA562645, and the analyses were conducted following a previous study.^[^ ^5^ ^]^

### siRNA‐Mediated Gene Knockdown

To modulate the expression of CCND1, Trim16, and Trim25 in VICs, cultured VICs at 70% to 80% confluency were transfected with specific siRNA or scrambled siRNA using Lipofectamine 3000 according to the manufacturer's instructions. si‐CCND1 and si‐Trim16 were designed and synthesized by GeneChem (Table S2, Supporting Information). After transfection, the medium was replaced with OM and subsequently used for further experiments.

### Real‐Time PCR

Total RNA from VICs was extracted using TRIzol reagent (Thermo Fisher), and the resulting supernatants were purified using the Direct‐zol RNA MicroPrep Kit (Zymo Research) following the manufacturer's instructions. The extracted total RNA was reverse transcribed using iScript Reverse Transcription Supermix (BIO‐RAD). The obtained cDNAs were used for reverse transcription‐PCR (RT‐PCR) experiments utilizing the ABI StepOnePlus System (Applied Biosystems, USA). The primers for RT‐PCR (Table S3, Supporting Information) were designed using Primer 5.0 software and synthesized by GenScript Co., Ltd (Nanjing, China). The data were analyzed using the 2^−ΔΔCt^ method.

### Statistical Analysis

All results were analyzed using SPSS 22.0 software (SPSS Inc., USA). Student's *t*‐test was employed for comparisons between two independent groups, while variance (analysis of variance) with Tukey's multiple comparisons test was used for comparisons involving three or more groups. Data were expressed as mean ± standard deviation, and a *p*‐value of <0.05 was considered statistically significant.

## Conflict of Interest

The authors declare no conflict of interest.

## Author Contributions

Z.L., K.W., C.J., and Y. C. contributed equally to this work. Z.L., K. W., and Y. W. conceived, designed, and supervised the experiments. C.J., Y.C., F.L., and M.X. performed the experiment. W.Y.Y, D.Y., X.Q., and S.C. analyzed the data and prepared figures. K.X., Y.W., N. D., and Z. L. wrote the final manuscript. Y.W., J. S., and N. D. provided the financial support. The authors declare that all data were generated in‐house and that no paper mill was used.

## Supporting information

Supporting Information

Supporting Information

## Data Availability

The data that support the findings of this study are available from the corresponding author upon reasonable request.
