# Supplementary material for: Morusin Alleviates Aortic Valve Calcification by Inhibiting Valve Interstitial Cell Senescence Through Ccnd1/Trim25/Nrf2 Axis
Source: Adv Sci (Weinh). 2024 Mar 19;11(20):2307319. doi: 10.1002/advs.202307319 (PMC11132047; doi:10.1002/advs.202307319)

ADVANCED SCIENCE
Open Access

Supporting Information

for *Adv. Sci.*, DOI 10.1002/advs.202307319

Morusin Alleviates Aortic Valve Calcification by Inhibiting Valve Interstitial Cell Senescence Through Ccnd1/Trim25/Nrf2 Axis

Zongtao Liu, Kan Wang, Chen Jiang, Yuqi Chen, Fayuan Liu, Minghui Xie, Wai Yen Yim, Dingyi Yao, Xingyu Qian, Shiqi Chen, Jiawei Shi, Kang Xu*, Yixuan Wang* and Nianguo Dong**

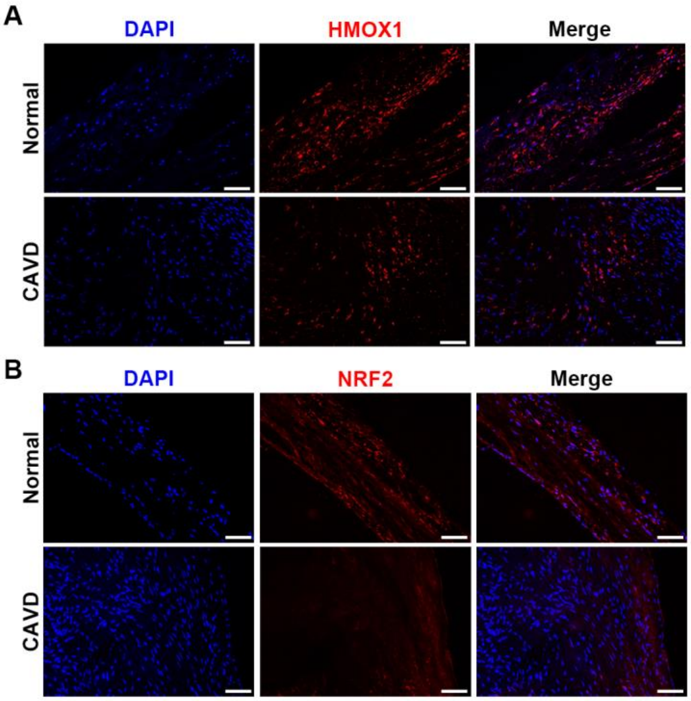

Figure S1. (A) Immunofluorescent staining of HMOX1 (red) and DAPI (blue) in the human aortic valve from CAVD patients and controls. Scale bar 100μm. (B) Immunofluorescent staining of NRF2 (red) and DAPI (blue) in the human aortic valve from CAVD patients and controls. Scale bar 100μm.

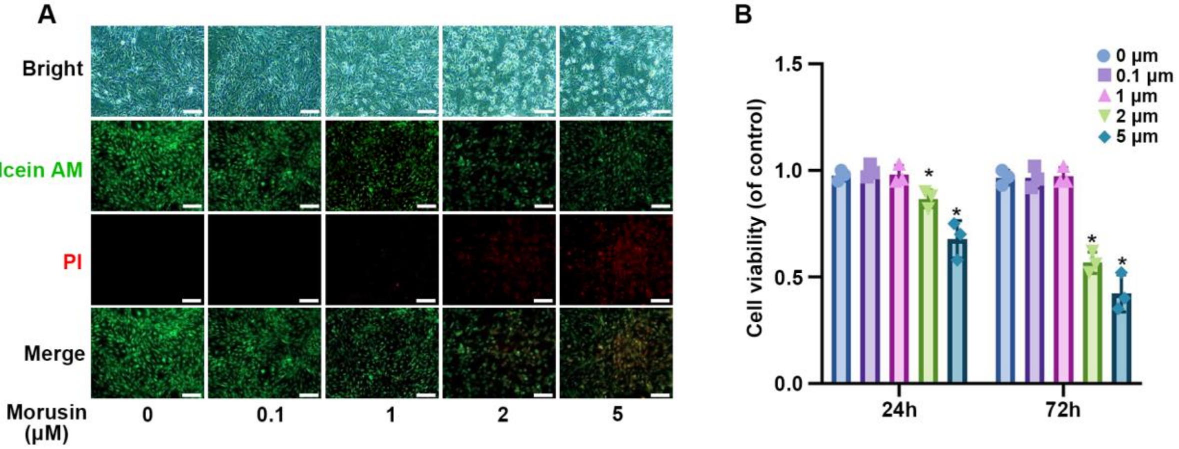

Figure S2. (A-B) Cell viability assessment of VICs with administration of different concentrations of morusin (n=3, each group). Data are means ± SD (n=3). Scale bar 50μm. Data are means ± SD. NS, not significant; *P<0.05; **P<0.01; ***P<0.001 (ANOVA with Tukey's multiple comparisons test).

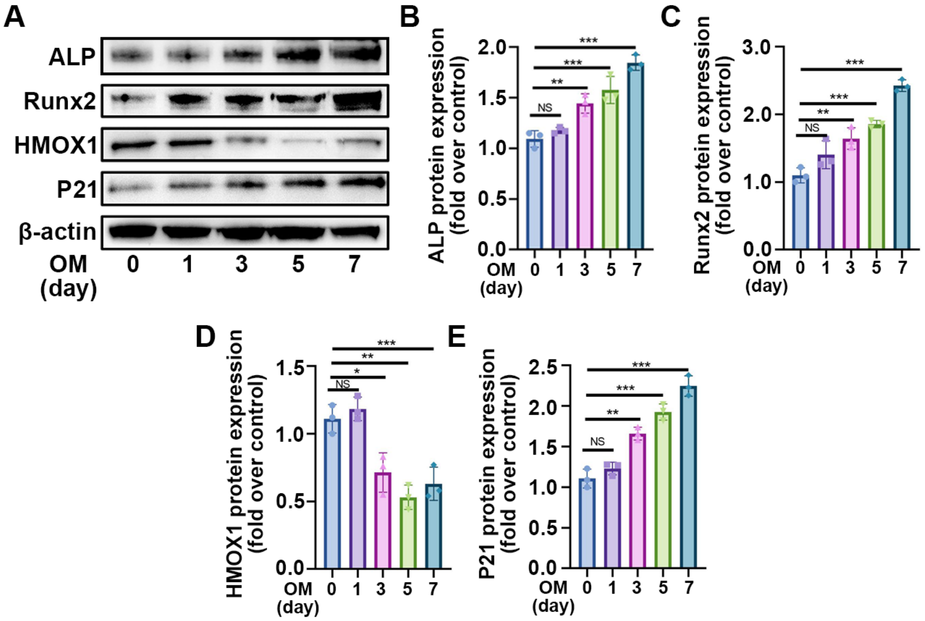

Figure S3. With OM induction for indicated time, immunoblot analysis of ALP, Runx2, HMXO1 and P21 expression in VICs (n=3, each group). Bar plots showing the semiquantitative analysis of indicated genes expression. Data are means ± SD. NS, not significant; *P<0.05; **P<0.01; ***P<0.001 (ANOVA with Tukey's multiple comparisons test).

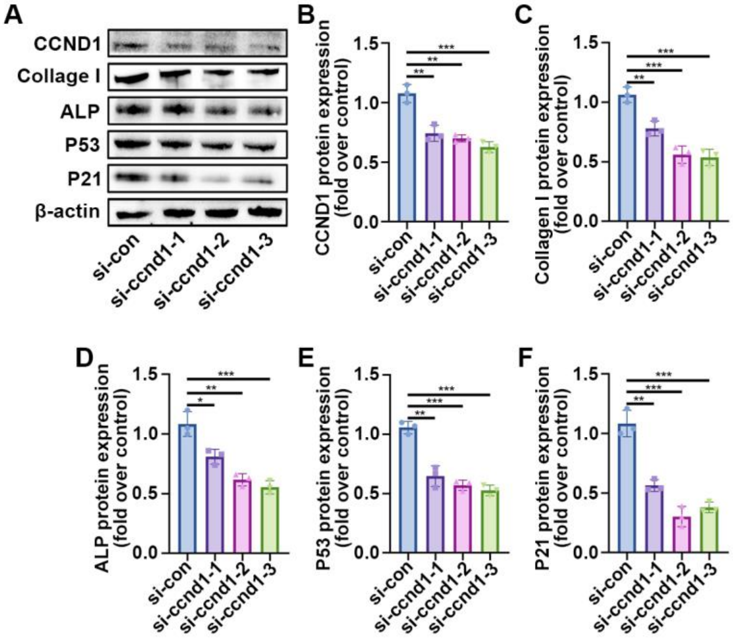

Figure S4. VICs were transfected with three different CCND1 siRNAs accordingly or scrambled siRNA, and then stimulated with OM for 7 days. Immunoblot analysis of CCND1, Collagen I, ALP, P53 and P21 expression in VICs from indicated groups (n=3, each group). Bar plots showing the semiquantitative analysis of indicated genes expression. Data are means ± SD. *P<0.05; **P<0.01; ***P<0.001 (ANOVA with Tukey's multiple comparisons test).

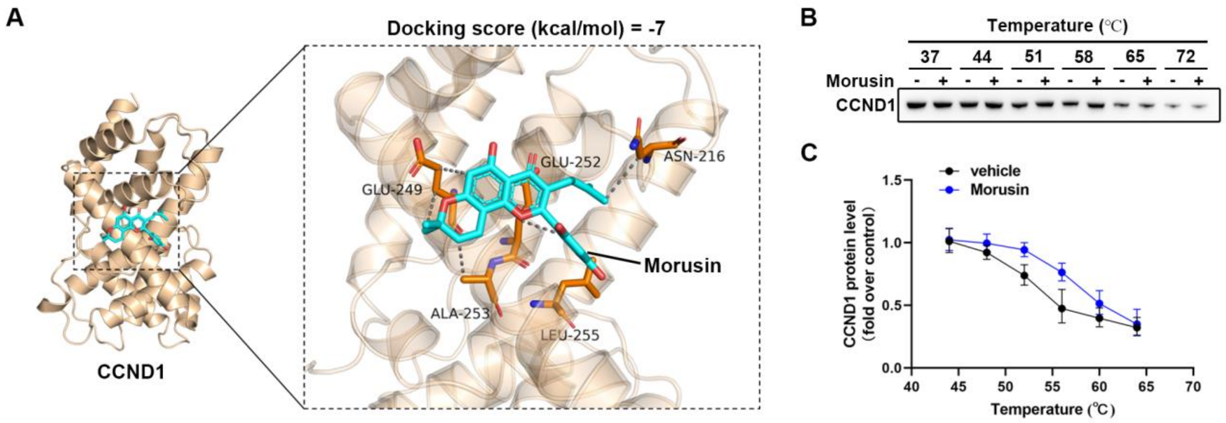

Figure S5. (A) Molecular docking model between CCND1 and morusin. (B-C) CETSA-WB assay to verify the direct interaction between CCND1 and morusin.

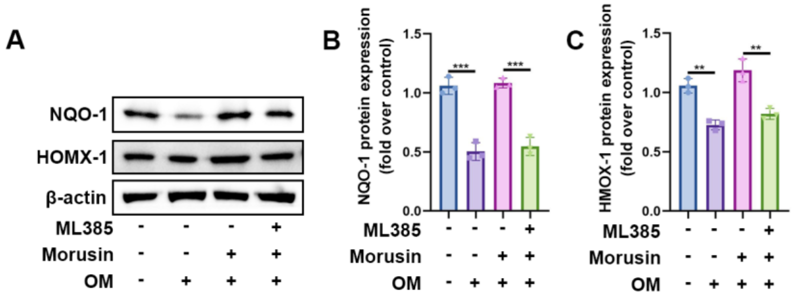

Figure S6. (A) ML385 was used to inhibit the activation of Nrf2 in VICs. Representative immunoblot images and quantification of the levels of NQO1 and HOMX-1 in VICs from indicated groups (n=3, each group). **P<0.01; ***P<0.001 (ANOVA with Tukey's multiple comparisons test).

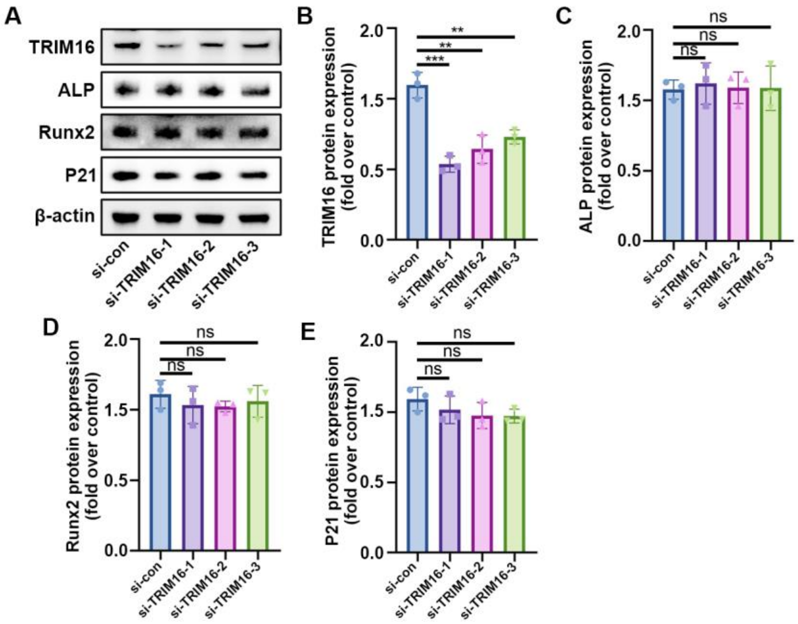

Figure S7. VICs were transfected with three different TRIM16 siRNAs accordingly or scrambled siRNA, and then stimulated with OM for 7 days. Immunoblot analysis of TRIM16, ALP, Runx2 and P21 expression in VICs from indicated groups (n=3, each group).**P<0.01; ***P<0.001 (ANOVA with Tukey's multiple comparisons test).

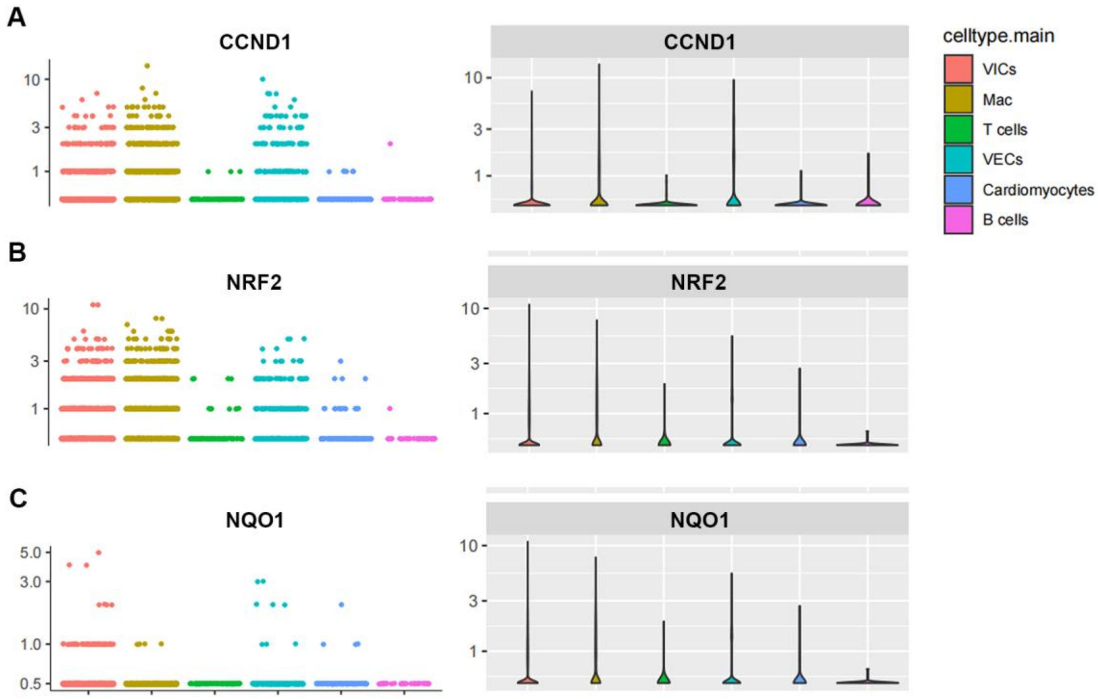

Figure S8. (A-C) The mRNA expression of CCND1, NRF2 and NQO1 in different cell types from mouse aortic valve tissue with single cell RNA sequencing.

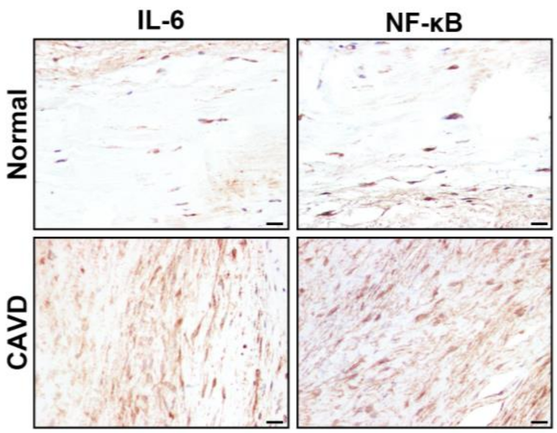

Figure S9. Representative immunohistology staining images of IL-6 and NF-κB in aortic valve tissue obtained from CAVD patients and their normal controls.

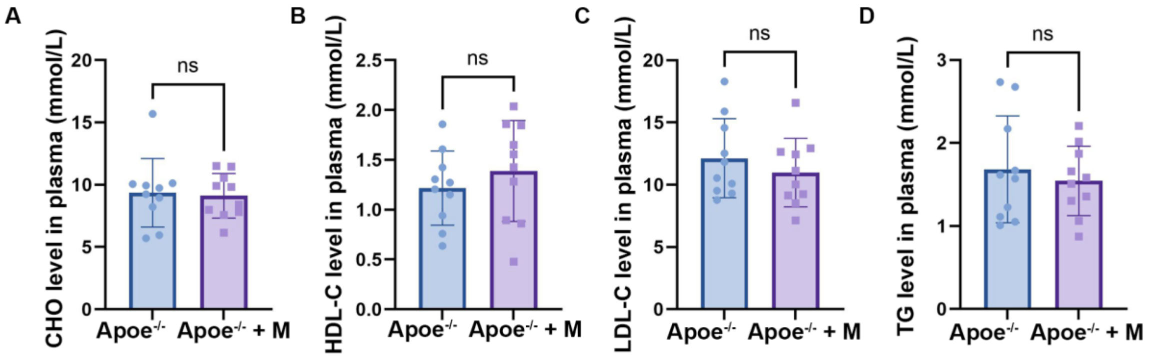

Figure S10. The measurements of LDL, CHO, TG and HDL in mice serum from indicated groups (n=10, each group). Ns, not significant (unpaired two-tailed Student's t test).

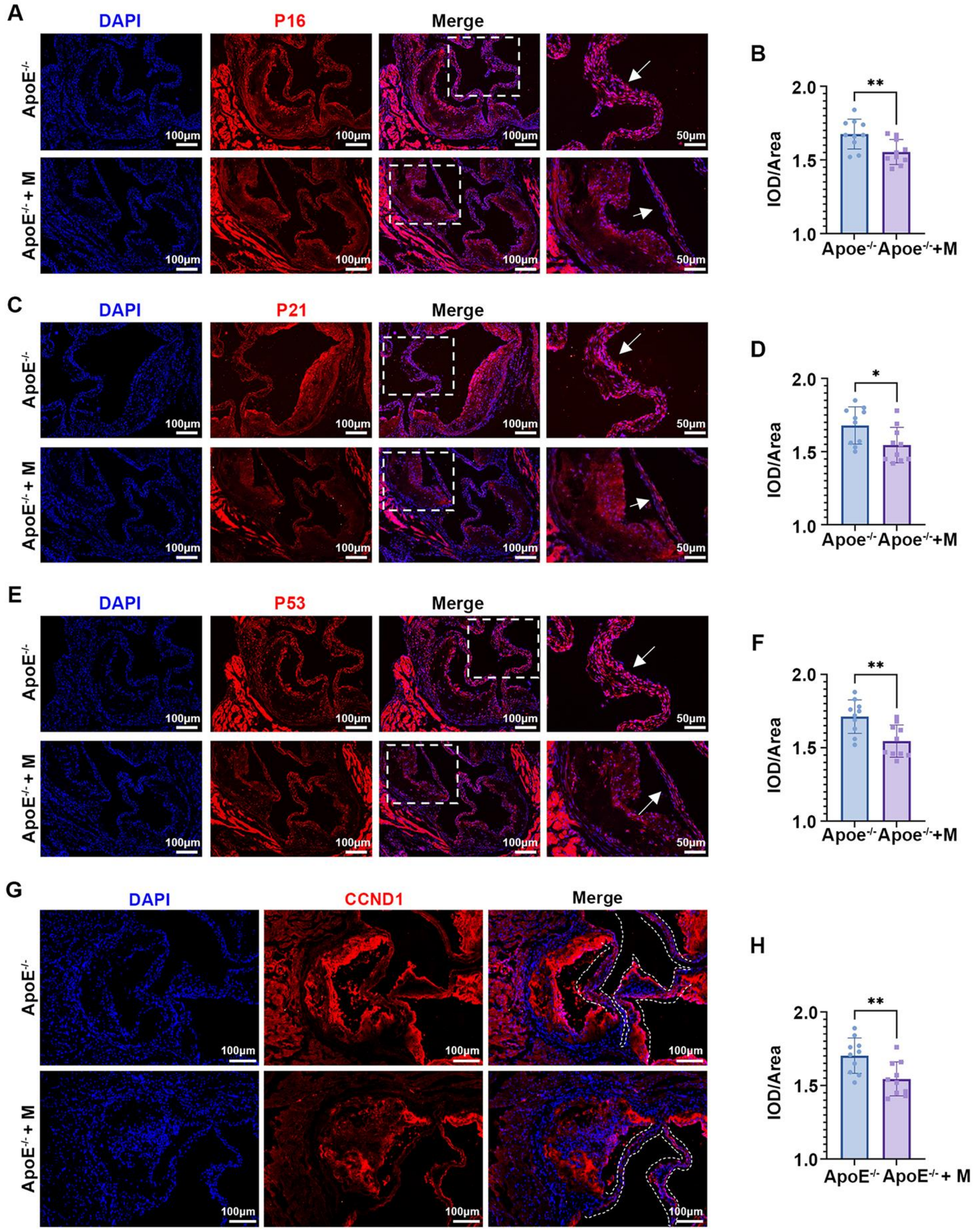

Figure S11. Immunofluorescent staining of P16 (A), P21 (C), P53 (E) and CCND1 (G) in the mouse aortic valve from indicated groups (n=10, each group). Bar plots showing the semiquantitative analysis of

fluorescence intensity of P16 (B), P21 (D), P53 (F) and CCND1 (H). Data are mean ± SD. *P<0.05; **P<0.01 (unpaired two-tailed Student's t test).

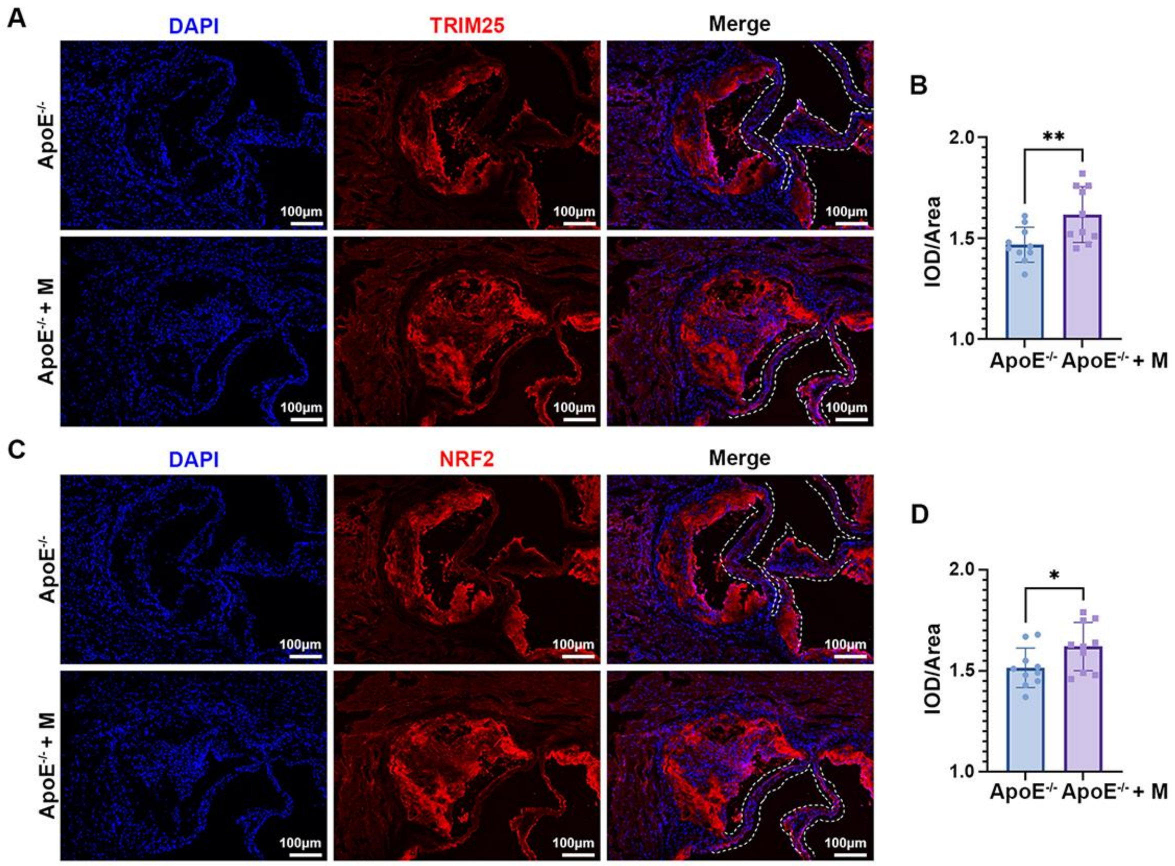

Figure S12. Immunofluorescent staining of TRIM25 (A) and NRF2 (C) in the mouse aortic valve from indicated groups (n=10, each group). Bar plots showing the semiquantitative analysis of fluorescence intensity of TRIM25 (B) and NRF2 (D). Data are mean ± SD. *P<0.05; **P<0.01 (unpaired two-tailed Student's t test).

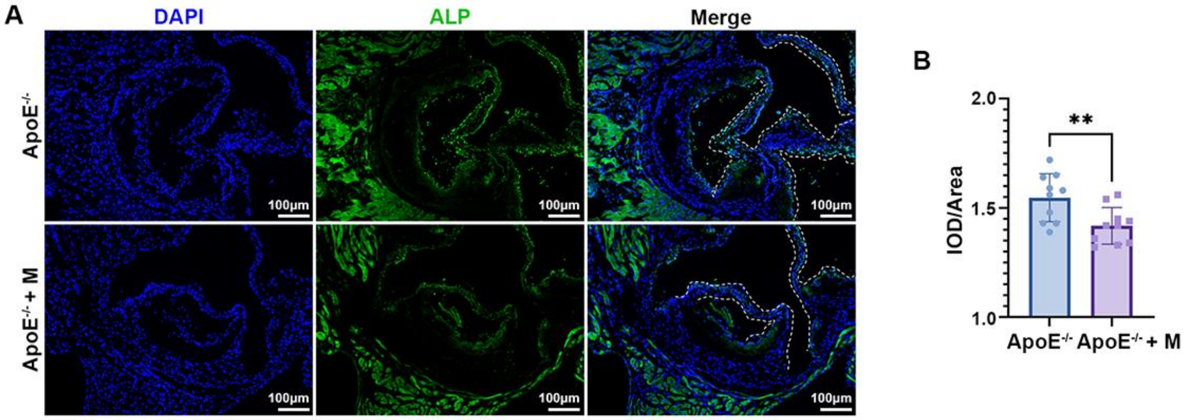

Figure S13. (A) Immunofluorescent staining of ALP in the mouse aortic valve from indicated groups (n=10, each group). (B) Bar plots showing the semiquantitative analysis of fluorescence intensity of ALP. Data are mean ± SD. *P<0.05; **P<0.01 (unpaired two-tailed Student's t test).

Table S1. Demographic and baseline clinical characteristics of Control and CAVD.

Note: Values are mean ± standard deviation (SD) or %

CAVD, calcific aortic valve disease; NS, nonsignificant; AV, Aortic valve; LVEF, left ventricular

	Control (n=10)	CAVD (n=10)	*P*
Mean age (years)	52.9 ± 4.1	55.8 ± 6.2	NS
Male/total	5/10	6/10	NS
Weight (kg)	67.1±6.7	70.7±3.7	NS
Hypertension	3/10	2/10	NS
Hypercholesterolemia	2/10	3/10	NS
Diabetes mellitus	1/10	3/10	NS
Smoking	3/10	4/10	NS
LVEF (%)	40 ± 11.7	56 ± 4.9	***

ejection fraction.（ *P <0 .05 , **P <0 .001 , ***P <0 .0001 ）

Table S2. Sequences (5′-3′) of siRNA for CCND1.

Homo si-CCND1-1	CCCGCACGATTTCATTGAA
Homo si-CCND1-2	TCGGTGTCCTACTTCAAAT
Homo si-CCND1-3	CCGAGAAGCTGTGCATCTA
Homo si-TRIM16-1	GCATCAGGTGAACATCAAA

Homo si-TRIM16-2	GCAGTGAAGTCCTGTCTAA
Homo si-TRIM16-3	GACCACAACTGGCGATACT

Table S3. Sequences (5′-3′) of oligonucleotide primers and probes. R, reverse; F, forward.

Homo CCND1-F	CCATCACCTGTCCTGTGTCTTCC
Homo CCND1-R	TCCTCAGCCTCCAGCCATCC

Table S4. Comparison of echocardiographic, and hemodynamic parameters in *Apoe*$^{-/-}$ mice of different groups.

Parameters	*Apoe*$^{-/-}$ control group (n = 10)	*Apoe*$^{-/-}$ +Morusin group (n = 10)
BW before, g	20.5 ± 1.5	22.2 ± 2.27
BW after, g	39.2 ± 1.24	38.1 ± 1.51
Heart rate, bpm	517.26 ± 33.19	513.3 ± 29.57
LVEDd, mm	3.95 ± 0.19	3.93 ± 0.28
FS, %	37.5 ± 6.7	37.2 ± 5.6
Aortic valve velocity, mm/s	1419 ± 224.5	1128 ± 165.1**

Note: Data are presented as mean ± standard deviation (SD).

Abbreviations: Apoe-/-, apolipoprotein E–deficient; BW, body weight; FS, functional shortening;

HCD, high cholesterol diet; LVEDd, left ventricular end-diastolic diameter; **P <0 .01.

Unedited gel for each representative cropped gel within the manuscript:

Figure 1F

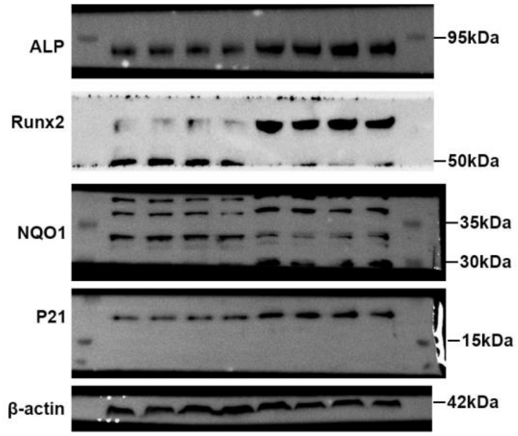

Figure 2A

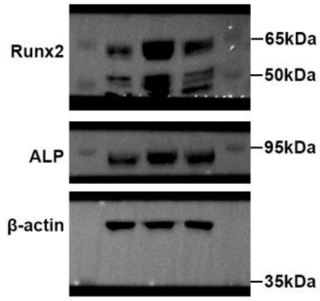

Figure 3K

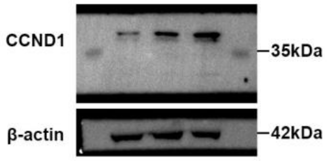

Figure 3E

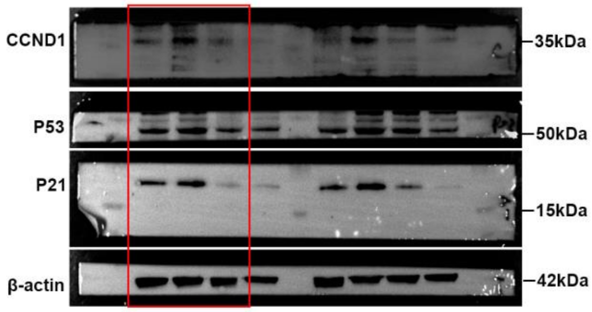

Figure 4A

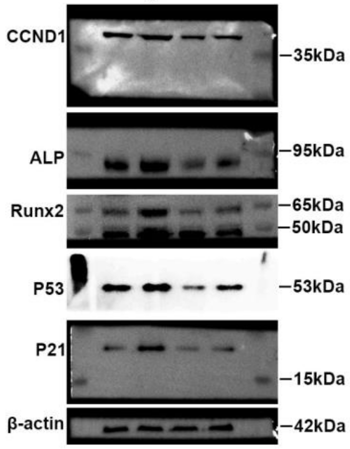

Figure 5A

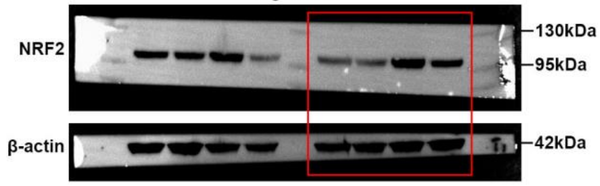

Figure 5C

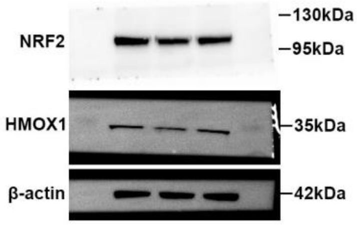

Figure 6A

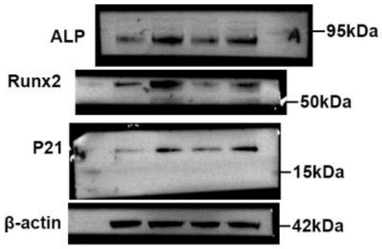

Figure 7B

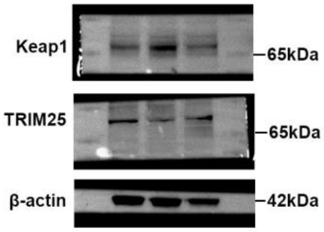

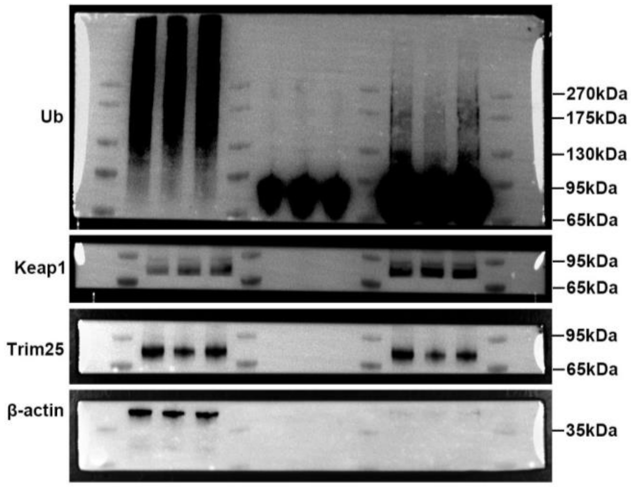

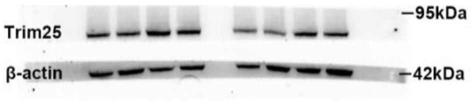

Figure 8C

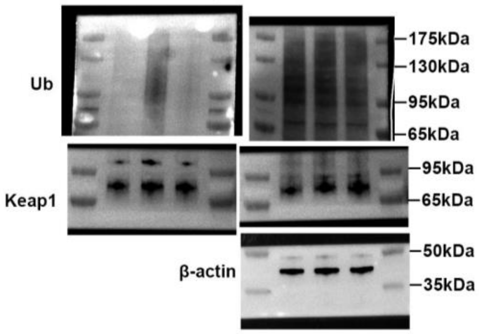

Figure 8D

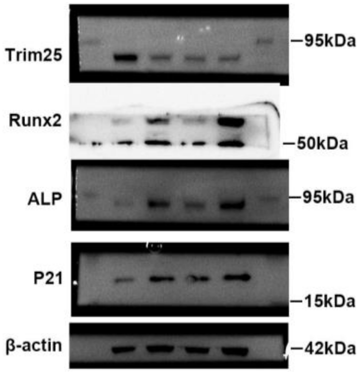

Figure 8I

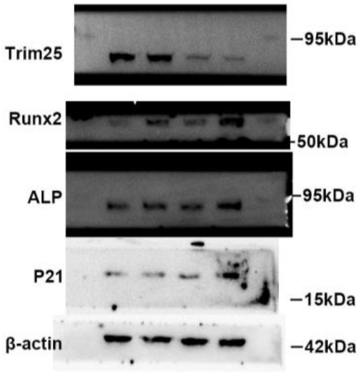

Figure S3

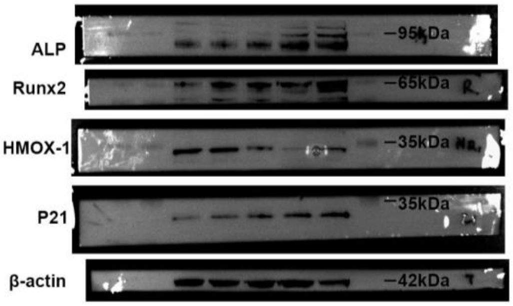

Figure S4

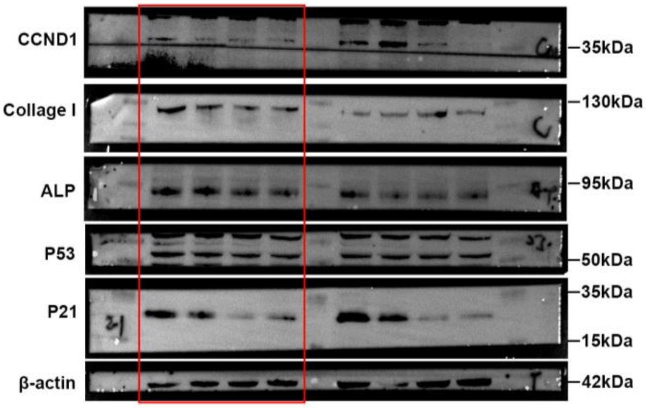

Figure S5

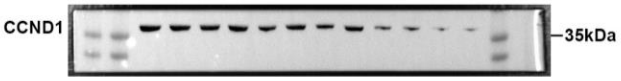

Figure S6

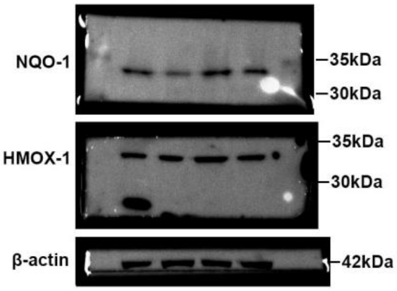

Figure S7

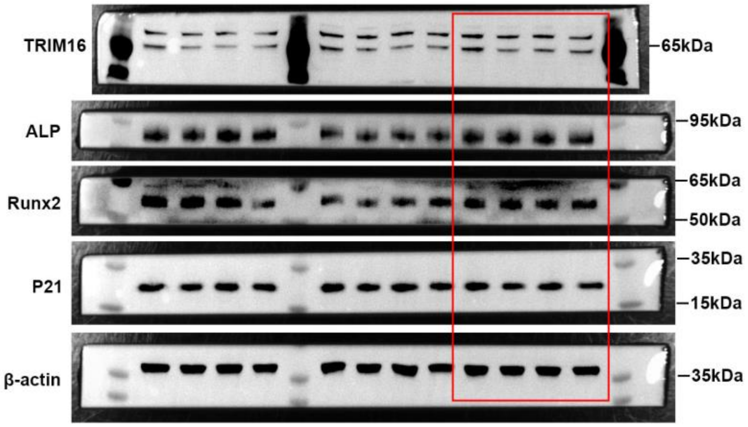

Supplement: Supplementary file 1 — Supporting Information [file ADVS-11-2307319-s001.pdf]
